# SNACS: a tool for demultiplexing single-cell DNA sequencing data

**DOI:** 10.1093/bioinformatics/btaf265

**Published:** 2025-06-05

**Authors:** Vanessa E Kennedy, Ritu Roy, Cheryl A C Peretz, Andrew Koh, Elaine Tran, Catherine C Smith, Adam B Olshen

**Affiliations:** Division of Blood and Marrow Transplantation and Cellular Therapy, Department of Medicine, Stanford University, Stanford, CA, 94304, United States; Hellen Diller Family Comprehensive Cancer Center, University of California San Francisco, San Francisco, CA, 94158, United States; Hellen Diller Family Comprehensive Cancer Center, University of California San Francisco, San Francisco, CA, 94158, United States; Division of Hematology and Oncology, Department of Pediatrics, University of California San Francisco, San Francisco, CA, 94143, United States; Division of Hematology and Oncology, Department of Medicine, University of California San Francisco, San Francisco, CA, 94143, United States; Division of Hematology and Oncology, Department of Medicine, University of California San Francisco, San Francisco, CA, 94143, United States; Hellen Diller Family Comprehensive Cancer Center, University of California San Francisco, San Francisco, CA, 94158, United States; Division of Hematology and Oncology, Department of Medicine, University of California San Francisco, San Francisco, CA, 94143, United States; Hellen Diller Family Comprehensive Cancer Center, University of California San Francisco, San Francisco, CA, 94158, United States; Division of Epidemiology and Biostatistics, University of California San Francisco, San Francisco, CA, 94143, United States

## Abstract

**Motivation:**

Single-cell DNA sequencing (scDNA-seq) and multi-modal profiling with the addition of cell-surface antibodies (scDAb-seq) have recently provided key insights into cancer heterogeneity. Scaling these technologies across large patient cohorts, however, is cost and time prohibitive. Multiplexing, in which cells from unique patients are pooled into a single experiment, offers a possible solution. While multiplexing methods exist for scRNAseq, accurate demultiplexing in scDNAseq remains an unmet need

**Results:**

Here, we introduce SNACS: single-nucleotide polymorphism and antibody-based cell sorting. SNACS relies on a combination of patient-level cell-surface identifiers and natural variation in genetic polymorphisms to demultiplex scDNAseq data. We demonstrated the performance of SNACS on a dataset consisting of multi-sample experiments from patients with leukemia where we knew truth from single-sample experiments from the same patients. Using SNACS, accuracy ranged from 0.948 to 0.991 versus 0.552 to 0.934 using demultiplexing methods from the single-cell literature.

**Availability and implementation:**

SNACS is available at  https://github.com/olshena/SNACS.

## 1 Introduction

Single-cell DNA sequencing (scDNA-seq) is an emerging microfluidic technology used in cancer research. Over the past several years, this technology has provided several key insights into cancer biology, intratumor heterogeneity, and clonal evolution (Lim *et al.* 2020, Ediriwickrema *et al.* 2023). Through direct measurement of mutational co-occurrence and acquisition, scDNA-seq can be used to reconstruct tumor phylogeny (Gawad *et al.* 2014, Jahn *et al.* 2016, Malikic *et al.* 2019, Miles *et al.* 2020, Morita *et al.* 2020), and serial measurements have further provided insight into treatment resistance and outcomes (McMahon *et al.* 2019, Peretz *et al.* 2021, Kennedy *et al.* 2022). Through precise genetic profiling, scDNA-seq also provides improved ability to detect low-level disease and can thus distinguish clinically meaningful residual disease from non-cancerous populations (Dillon *et al.* 2021, Ediriwickrema *et al.* 2023). More recently, scDNA-seq has been combined with single-cell measurements of cell-surface protein expression in a technology known as “scDAb-seq” for SC DNA and Antibody-seq (Miles *et al.* 2020, Morita *et al.* 2020, Demaree *et al.* 2021). This multi-omic technology provides novel insight into the complex relationship between cancer genotype and phenotype (Miles *et al.* 2020, Morita *et al.* 2020, Demaree *et al.* 2021). Taken together, scDNA-seq and scDAb-seq have opened a new frontier in cancer research.

Despite these abilities, there are several limitations to using these technologies at scale. Both scDNA-seq and scDAb-seq are costly (Ediriwickrema *et al.* 2023) in terms of material and time needed to perform single cell assays, restricting adoption to highly resourced research laboratories. To date, most scDNA-seq studies on human samples have included fewer than 10 patients and analysis of large patient cohorts and/or multiple timepoints per patient remains cost prohibitive. These costs limit the translation of single-cell technologies from research to viable clinical assays (Lei *et al.* 2021).

One strategy for mitigating such challenges is *multiplexing*, in which cells from multiple unique individuals are pooled into a single microfluidic run and then subsequently *demutiplexed* into their constitute components using bioinformatic tools. If used successfully, this strategy can result in lower per sample library preparation costs and increased efficiency, albeit with the tradeoff of a lower number of single cells captured per sample. Multiplexing can also be highly error prone. In addition to incorrectly assigning cells to parent samples, multiplexing can result in *multiplets* where single cells from two or more individuals are encapsulated into a single droplet causing information from multiple individuals to be falsely associated with a single cell barcode. Without accurate identification and removal, multiplets may incorrectly appear to be unique cell populations and thus lead to inaccurate downstream analyses.

**Figure 1.**
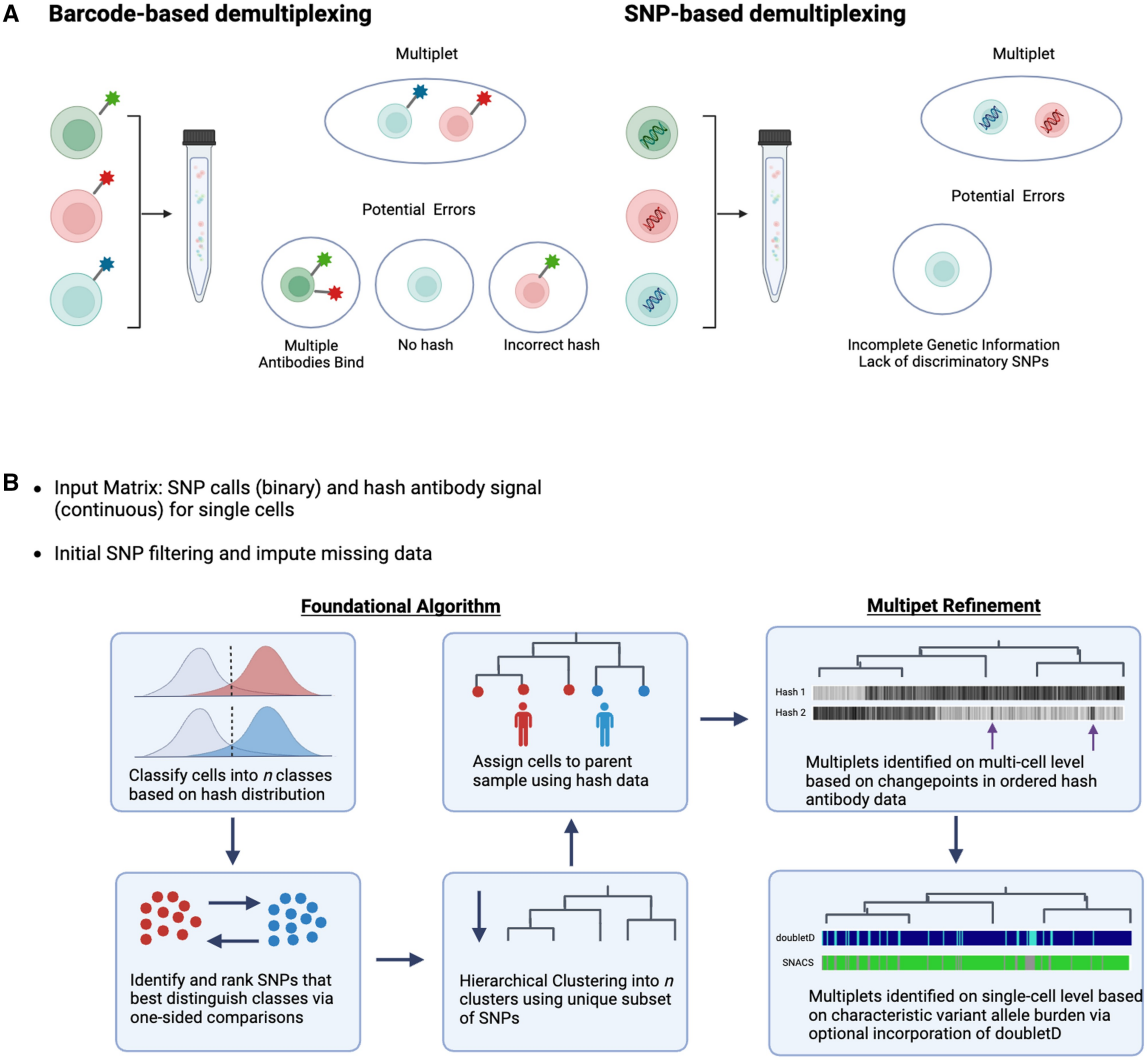
(A) Existing demultiplexing approaches described in scRNA-seq include barcode- and SNP-based approaches. Both are imperfect and require identification of multiplets. (B) Schematic of SNACS algorithm. SNACS offers a novel, combinatory approach using both SNPs and barcoded “hash” antibodies to demultiplex samples and resolve mutiplets.

To date, single-cell multiplexing and multiplet identification has primarily been described in the single-cell RNAseq literature. Methods include barcode-based and single nucleotide polymorphism (SNP)-based approaches (Fig. 1A). In barcode based approaches, cells from unique samples are labeled with sample-level DNA barcodes and attached either via cell-surface antibodies (Stoeckius *et al.* 2018, Kim *et al.* 2020), lipid-bound cell membrane tags (McGinnis *et al.* 2019), or viral integration of DNA barcodes directly into the genome (Guo *et al.* 2019). In SNP-based approaches, multiplexed samples are demultiplexed based on natural genetic polymorphisms or “endogenous” barcodes (Kang *et al.* 2018, Huang *et al.* 2019, Xu *et al.* 2019, Heaton *et al.* 2020). Both strategies have limitations.

In barcode-based multiplexing, cells may be bound by multiple different sample-level barcodes, the incorrect barcode, or no barcode entirely. In SNP-based demultiplexing, multiplexed samples may be unclassifiable if sufficient discriminatory SNPs are not present or if sequencing depth is inadequate. Importantly, SNP-based demultiplexing is also dependent on *a priori* knowledge to assign cells to their sample of origin.

In this work, we describe a novel scDNA-seq multiplexing approach, algorithm, and visualization methodology. Based upon DAbseq technology, this approach combines both SNP and barcoding based approaches for demultiplexing and multiplet identification, thus increasing accuracy. We have called this approach SNACS, for **SN**P and **A**ntibody-based **C**ell **S**orting. Our formulation is novel because previously, as far as we know, SNP and barcoding information have not been used in tandem for demultiplexing.

## 2 Materials and methods

### 2.1 Algorithm 

The SNACS algorithm described in detail here is outlined in Fig. 1B. For each multiplexed experiment, SNPs are treated as binary (mutated or wildtype) and hash antibody expression is treated as continuous. SNP data are first filtered to remove both SNPs and single cells with high missingness. We used a threshold of 40% missing data for both, but results should not be sensitive to these choices. Hash antibody counts are normalized using the centered log ratio transformation, as is common in both SC DAbseq and CITEseq analysis (Mule *et al.* 2022). In this normalization, the hash antibody count for every cell is divided by the geometric mean across cells and then log base 2 transformed.

#### 2.1.1 Hash antibody data provides initial classification

First, hash antibody data is used to classify cells into *n* preliminary groups, where *n* is the number of parent samples and thus hash antibodies. For each hash antibody, the antibody expression for a multiplexed experiment is expected to be bimodal, with one right mode comprised of antibody-stained cells belonging to a single parent sample and one left mode comprised of unstained cells from alternate parent samples.

To estimate the background antibody distribution, we generate a symmetric distribution by reflecting the data to the left of the left mode about that mode (Fig. S1, available as supplementary data at *Bioinformatics* online). Cells are assigned to a specific hash antibody if the antibody expression of that cell is expressed highly; we used a threshold of the 95th percentile of the background distribution to estimate a cell as positive for a hash. If fewer than 100 cells are assigned to any parent sample, then a more robust approach is used to estimate the background antibody distribution. If only one mode is initially found within the antibody distribution, then the bandwidth for background estimation is decreased by 25% until two modes are found. Similarly, if more than two modes are found initially, then the bandwidth is decreased by 25% until two modes are found. Cells that were assigned to either multiple hashes or to no hashes were excluded in this step.

#### 2.1.2 SNPs that separate preliminary groups are identified

Next, we select the SNPs that best distinguish the hash-antibody-defined groups from the initial preliminary classifications of the previous step. For each SNP, we compare the proportion of mutated (i.e. 1) values pairwise between hash groups and rank these comparisons using a one-sided chi-square test. Our test is one-sided to identify SNPs that have a higher proportion of positivity for each comparison. For each of *n* groups, *n* −* 1* comparisons are performed, and for each comparison, we choose the top *k* SNPs. The value of *k* is user-defined and we have set the default as three. The result of our comparisons identifies a total of n*(*n* − 1)*k SNPs that separate groups, but as the same SNPs may be chosen for more than one comparison, we limit ourselves to the unique subset of SNPs.

#### 2.1.3 SNPs are hierarchically clustered and hash antibody data refines those clusters and assigns them to parent samples

Next, we perform agglomerative hierarchical clustering of the binary SNP data from all cells using the unique subset of SNPs identified in the previous step. Prior to clustering, missing SNP data is imputed utilizing a majority vote of the five nearest neighbors from the kNN function of the VIM R Package. Clustering is performed using cosine as the distance function and Ward’s method for joining clusters. The resulting dendrogram is cut into *n* clusters. Clusters are further identified by traversing down the hierarchical tree and splitting if a significant difference is found when comparing the daughter nodes of any current node. The comparisons are made for every hash antibody, and the two-sample *t*-test with equal variances is used to test for differences with a default unadjusted *P*-value threshold of 10^−5^. The process is stopped when no additional differences are found. We do not allow clusters of fewer than two cells.

Clusters are then assigned to a specific hash antibody and parent sample by comparing the antibody expression of the cluster to the hash background distributions as described in Section 2.1.1. In our analysis we assigned a cluster to a hash if > 50% of cells from that cluster have a hash expression that exceeded the 95th percentile of the background distribution. Clusters assigned to multiple hashes are designated as multiplets, and clusters not assigned to a hash antibody are designated “no call.” In the SNACS R package, the output and visualization of this initial demultiplexing is designated “SNACS Round 1” (Figs S2–S4, available as supplementary data at *Bioinformatics* online).

#### 2.1.4 Refined multiplet detection using circular binary segmentation

As accurately detecting multiplets is a significant concern in multiplexed SC data, we perform up to two additional steps to improve multiplet detection. First, we refined the calling of multiplets at the multi-cell level using hash antibody data. To do this, we estimate the mean hash antibody expression for each hash based on the cell clusters that were uniquely assigned to that hash in SNACS Round 1 (Section 2.1.3). Then, we calculate the Euclidean distance to the cluster center for every cell and hash. Within each cluster, using the ordering of the cells determined by the clustering, we next segment the distance value for every hash by using circular binary segmentation (CBS), an algorithm designed to identify contiguous regions of homogenous DNA copy in the genome by estimating changepoints in sequential data (Olshen *et al.* 2004). The superset of all changepoints is then used to form new clusters, and those new clusters are then, possibly, reassigned in multiplets using the same approach as described in Section 2.1.3.

In this analysis, only narrow segments are considered; we chose 100 or fewer cells as narrow. To provide additional power in this step, our hash background cutoff was the 75th percentile instead of the 95th. In the SNACS R package, the output and visualization of this optional subsequent refinement step is designated “SNACS Round 2” (Figs S2–S4, available as supplementary data at *Bioinformatics* online).

#### 2.1.5 Additional multiplet detection via combination with doubletD

We also include the capability to call multiplets at the single-cell level by incorporating the previously published doubletD algorithm (Weber *et al.* 2021). In doubletD, matrices of total and alternate allele depth for each single-cell barcode are used to identify doublets based on increased allele frequency and/or drop-out via an expectation-maximization approach. For each single cell in our multi-sample experiments, we considered the cell a multiplet if it was called a multiplet by SNACS, as detailed above, *or* by doubletD. In the SNACS R package, the output and visualization of this optional subsequent refinement step is designated “**SNACS plus doubletD**.” Of note, although the mathematics of the doubletD algorithm are identical to those previously published, we translated the author-supplied code from python to R for seamless incorporation into our software.

##  

### 2.2 Generation of empiric data from leukemia patient samples

To evaluate the SNACS algorithm, we generated empiric data by conducting eleven scDAb-seq experiments on combinations of pooled samples from eight adult patients with acute myeloid leukemia using a microfluidic approach via the Tapestri platform (MissionBio). The first seven experiments were used to generate our core empiric data from which our algorithm was built, and the remaining four experiments were used to generate our validation data. Briefly, cryopreserved cells were thawed, normalized to 10 000 cells/μL in 180 μL PBS (Corning), and then incubated with Human TruStain FcX (BioLegend) and salmon sperm DNA (Invitrogen) for 15 min at 4C. Cells were then incubated with oligo-conjugated cell-surface antibodies (TotalSeq “hashtag” antibodies, BioLegend) for 30 min to provide patient-level identifiers. Each patient was assigned a unique hash antibody, and patients were pooled together as described in Tables 1 and 2.

**Table 1.** Experimental plan for generation of empiric single-cell DNAseq data.

Experiment	Patient	Hash
Experiment 1	Patient A	TS-1
	Patient A	TS-2
Experiment 2	Patient B	TS-2
	Patient B	TS-3
Experiment 3	Patient C	TS-3
	Patient C	TS-4
Experiment 4	Patient D	TS-1
	Patient D	TS-4
Experiment 5	Patient A	TS-1
	Patient B	TS-2
Experiment 6	Patient B	TS-2
	Patient C	TS-3
	Patient D	TS-4
Experiment 7	Patient A	TS-1
	Patient B	TS-2
	Patient C	TS-3
	Patient D	TS-4

**Table 2.** Experimental plan for generation empiric validation single-cell DNAseq data.

Experiment	Patient	Hash
Experiment 8	Patient E	TS-5
	Patient F	TS-6
Experiment 9	Patient E	TS-5
	Patient F	TS-6
	Patient G	TS-7
	Patient H	TS-8
Experiment 10	Patient A	TS-1
	Patient B	TS-2
	Patient C	TS-3
	Patient D	TS-4
	Patient E	TS-5
	Patient F	TS-6
	Patient G	TS-7
	Patient H	TS-8
Experiment 11	Patient A	TS-1
	Patient B	TS-2
	Patient C	TS-3
	Patient D	TS-4
	Patient E	TS-5
	Patient F	TS-6
	Patient G	TS-7
	Patient H	TS-8

Next, pooled samples were resuspended in cell buffer (MissionBio), diluted to 4-7e6 cells/mL, and loaded onto a microfluidics cartridge, where individual cells were encapsulated, lysed, and barcoded using the Tapestri instrument. DNA from barcoded cells was amplified via PCR using a targeted panel that included 288 amplicons across 66 genes associated with acute myeloid leukemia (Table S1, available as supplementary data at *Bioinformatics* online). DNA PCR products were isolated, purified with AmpureXP beads (Beckman Coulter), used as a PCR template for library generation, and then repurified with AmpureXP beads. Protein PCR products from hash antibodies were isolated from the supernatant via incubation with a 5’ Biotin Oligo (ITD). Protein PCR products were then purified using Streptavidin C1 beads (Thermo Fisher Scientific), used as a PCR template for library generation, and then repurified using AmpureXP beads. Both DNA and protein libraries were quantified and assessed for quality via a Qubit fluorometer (Life Technologies) and Bioanalyzer (Agilent Technologies) prior to pooling for sequencing on an Illumina Novaseq.

FASTQ files were processed via an open-source pipeline as described previously (Demaree *et al.* 2021). Valid cell barcodes were called using the inflection point of the cell-rank plot in addition to the requirement that 60% of DNA intervals were covered by at least eight reads. Variants were called using GATK (v 4.1.3.0) according to GATK best practices (DePristo *et al.* 2011).

### 2.3 Estimation of accuracy

To test the accuracy of SNACS and other demultiplexing algorithms, we compared multi-sample experiments (Experiments 5–7) to single-sample experiments (Experiments 1–4). Our rationale was that the SNP profiles of the single-sample experiments would be reflected in the multi-sample experiments, thus allowing for an estimation of “truth.” Specifically, for each single cell in a multi-sample experiment, we assigned a “truth call” for each single cell by comparing the SNP profile of that cell against the SNP profile of the constituent single-sample experiments. We considered only SNPs that were genotyped in both the multi-sample and constituent single-sample experiments. Figures S5–S7, available as supplementary data at *Bioinformatics* online provides visualization of accuracy calculations and assessments.

We determined truth calls as follows. In the multi-sample experiments, we considered a single cell a true “singlet,” that is belonging to a specific single sample, when the SNP profile for non-missing SNPs, both positive and negative, exactly matched those same SNPs present in >0.5% of cells in a constituent single-sample experiment, was inconsistent for at least one SNP with other single-sample experiments, and could not be reconstructed from multiple samples. A single cell was called a “multiplet” when it was not a singlet and the non-missing SNP profile could be precisely reconstructed, without errors, from multiple single-sample experiments. Specifically, if one candidate single-sample was positive for a particular SNP, then the multiple cell must also be positive for that SNPs. A single cell was called “ambiguous” when it was not a singlet or a multiplet, or when a cell in a multi-sample experiment was comprised of a SNP profile in which a positive SNP was not adequately genotyped (i.e. was missing data) in the constituent single cell experiments.

For each of the three multi-sample experiments, we estimated total accuracy as the proportion of truth calls that were matched by the demultiplexing algorithm. We also developed measures of sensitivity and specificity to characterize how well a demultiplexing algorithm identified multiplets. Sensitivity was defined as the proportion of true multiplets that were called multiplets and specificity as the proportion of true singlets that were called singlets. We chose to define our metrics using this approach as sensitivity and specificity most commonly reflect the abnormal (or “disease”) state in biomedical sciences, and multiplets represent the abnormal state in multi-sample experiments. We additionally calculated the proportion of singlets assigned to an alternative single sample.

### 2.4 Comparison against alternative multiplexing approaches

Using our benchmarking dataset, we evaluated SNACS against the following five diverse multiplexing approaches:

1. **HTOdemux** is a barcode-based approach from the Seurat package developed to demultiplex cells in scRNA-seq experiments based on k-medoid clustering of cell-surface hash antibody values (Stoeckius *et al.* 2018). We used this method with default parameters and without modifications.
2. **CiteFuse** is a barcode-based approach developed to demultiplex cells in scRNA-seq experiments using a Gaussian mixture model fit to log-transformed cell-surface hash antibody values (Kim *et al.* 2020). We used this method with default parameters and without modifications.
3. **scSplit** is a SNP-based approach developed to demultiplex scRNA-seq data using initial k-means clustering followed by an expectation-maximization approach (Xu *et al.* 2019). To modify this approach to scDNA-seq data, we used this method starting with an allele count matrix of SNPs x single-cell barcodes. After this modification we used the method as constructed.
4. While there are currently no named algorithms for demultiplexing scDNA-seq data, a SNP-based approach is described in a publication by Robinson *et al.* (2023). This method uses initial k-means clustering followed by additional multiple identification by generating artificial multiplets and comparing them to true cells (Robinson *et al.* 2023). We refer to this method as the “Robinson method.”
5. **doubletD** is a SNP-based approach for detecting doublets in scDNA-seq data using an expectation-maximization approach, and is based on the observation the scDNA-seq multiplets tend to have an increase in number of allele copies and/or drop-out (Weber *et al.* 2021). doubletD only detects whether a cell is a doublet versus not a doublet and does not demultiplex single cells or assign them to parent samples. Thus, in our comparison to doubletD, we compared only its ability to accurately identify a multiplet.

### 2.5 Generation of simulated data for modeling effect of number of multiplexed samples, proportion of multiplets, number of input cells, and number of multiplexed samples

To model the ability of SNACS to demultiplex experiments with variable proportions of multiplets, number of input cells, and number of multiplexed samples, we simulated multiplexed data using Experiments 1–4, in which patient samples were run as single-sample (e.g. not multiplexed), yet still tagged with cell-surface antibodies. In these experiments, cells were split approximately in half with each half tagged with a different cell-surface hash antibody (Table 1). For these simulations, only the subset of cells with higher measurements for TS-1, TS-2, TS-3, and TS-4 hash antibody measurements were retained from Experiments 1–4, respectively.

Next, cells that were kept from Experiments 1–4 were merged to generate new datasets mimicking Experiments 5–7, with the same hash antibodies as the original experiments. For example, the remaining cells from Experiment 1 and Experiment 2 were merged to mimic Experiment 5. From these three new datasets, cells were sampled to obtain both singlets and novel multiplets, in which *C* is total number of sampled cells and *p* is proportion of multiplets:

1. **Singlets:** Singlet cells were sampled with replacement from the pseudo-experiments.
  $$C_{\mathrm{singlets}}=(1—p)\cdot C$$
2. **Multiplets:** Multiplet cells were generated by sampling ***C*_multiplets_ = *p***· ***C*** pairs of cells. The first cell was sampled from retained cells in Experiments 1–4. The second cell was sampled from the pseudo-experiment and the origin of the two cells could not be the same.

The genotypic variant calls for sampled cells were determined as follows. For singlets, variant calls were directly assigned based on the original cell's data. For multiplets, variant calls for each pair were combined according to the following rules: 1_cell_1_,1__cell2_ = 1_multiplet_; 0_cell_1_, 0_cell_2_ = 0_multiplet_; 0_cell_1_,1_cell_2_ = 1_multiplet_; 1_cell_1_, NA_cell_2_ = 1_multiplet_; 0_cell_1_, NA_cell_2_ = 0_multiplet_; NA_cell_1_, NA_cell_2_ = 0_multioplet_ in which 1 represents a mutated variant, 0 represents a wildtype variant, and NA represents a non-genotyped variant.

Next, we modeled hash antibody distributions for our pseudo-experiments. For each single cell, we assigned a *proper hash antibody*, or a positive hash antibody corresponding to that cell’s true origin, and *improper hash antibodies*, or hash antibodies that are a component of that experiment, but do not belong to that single cell. For each hash antibody *H*, the mean (μ_H_) and variance (σ^2^_H_) of proper hash antibodies were calculated from Experiments 1–4. Both proper and inproper hash signals in the simulated datasets were adjusted to ensure identical means and variances across proper and improper distributions: ***H*_adjusted_ = *a ⋅ H *+* b***, where *a* and *b* are constants determined to equalize means and variances. For pseudo-experiments with *S *=* *3 or *S *=* *4 multiplexed samples, Guassian noise **N(0, 0.5⋅ σ^2^_H_)** was added to the improper hash antibodies to prevent the distributions from becoming identical. For doublets, the mean of the proper hash antibody signals was decreased by 20%, and the variance was increased by one-third, based on empirical observations from Experiments 5–7:

The above approach was repeated for the following parameters: *S *=* *2, 3, and 4 samples multiplexed; *C *=* *2000, 4000, 6000, 8000, and 10 000 input cells; *p *=* *0.0, 0.05, 0.10, 0.15, 0.20, and 0.25 proportion multiplets. For each of these 90 possible combinations (*S* ***⋅*** *p* ***⋅*** *C*), 100 simulated datasets were generated. These datasets were demultiplexed using SNACS and other comparison methods. Performance metrics, including accuracy, sensitivity, and specificity, were calculated based on the ground truth of the cell’s origin.

### 2.6 Generation of simulated data for modeling effect of number of multiplexed samples

In our empiric experiments, corresponding single-sample data was available for experiments with up to 4 patients multiplexed together. Therefore, our above simulations only evaluated the ability of SNACS and other methods to demultiplex 2, 3, and 4 pooled samples. To model the ability of SNACS to demultiplex an increasing number of multiplexed samples, we performed a second simulation to evaluate the probability of observing a minimum number of cells in a sample as a function of the number of samples being multiplexed (*S*). The simulation included the following components:

1. **Number of Input Cells:** The total number of input cells (*C)* was simulated from a truncated Gaussian distribution based on the average number of evaluable input cells from our empiric data:
  $$C\;∼\text{Gaussian}\left(5300,{\text{ 2500}}^{2}\right),\text{C}\geq \text{ 15}00$$
2. **Proportion of Multiplets:** The proportion of cells lost as multiplets (*P_m_*) was determined by applying least squares to our empiric data and modeled as a linear function of *S:*
 
  $$P_{m}=0.1+0.25S$$

In the simulations, *P_m_* was drawn from a Gaussian distribution with variance taken from the error term of that linear model:

$$\;P_{m}∼\;N\left(0.1+0.25S,\;0.067^{2}\right)$$

1. **Lowest Proportion of Cells in Sample:** The smallest proportion of cells in a demultiplexed sample (*P*min) was similarly modeled using least squares, with the minimum proportion of cells set to 0.02.
  Pmin=max(0.33–0.04S, 0.02)

In our simulation values were sampled from:

$$P\text{min }∼\text{ N}\left(\text{max}\left(0.33–0.4S,\;0.02\right),\;0.070^{2}\right)$$

1. **Simulated Output:** For each simulation, the minimum number of cells in a demultiplexed sample (*C*_min_) was calculated as:
  $$C_{\text{min}}=C\cdot \left(1—P_{m}\right)\;\cdot {\;P}_{\min}$$

This value was compared against thresholds of 100–500 cells based on biologically significant thresholds described in the leukemia literature (Miles *et al.* 2020, Kennedy *et al.* 2022, Ediriwickrema *et al.* 2023). Simulations were repeated 10 000 times for multiplexing between two and eight samples.

### 2.7 Implementation details

SNACS software and associated documentation is freely available at https://github.com/olshena/SNACS. Version 1.0.3.4 was used in this analysis. Code used to generate truth calls and accuracy assessments is available as well at https://github.com/olshena/SNACS/tree/master/analysis. An explanation of how all R scripts are related is available at https://github.com/olshena/SNACS/blob/master/analysis/Analysis_README_Description_of_scripts.md. The data underlying this article (unprocessed FASTQ files and SNP and antibody matrices following FASTQ alignment and variant calling) are available in NCBI’s Gene Expression Omnibus (accession number GSE255224).

## 3 Results

### 3.1 Performance of SNACS on empiric patient data

**Figure 2.**
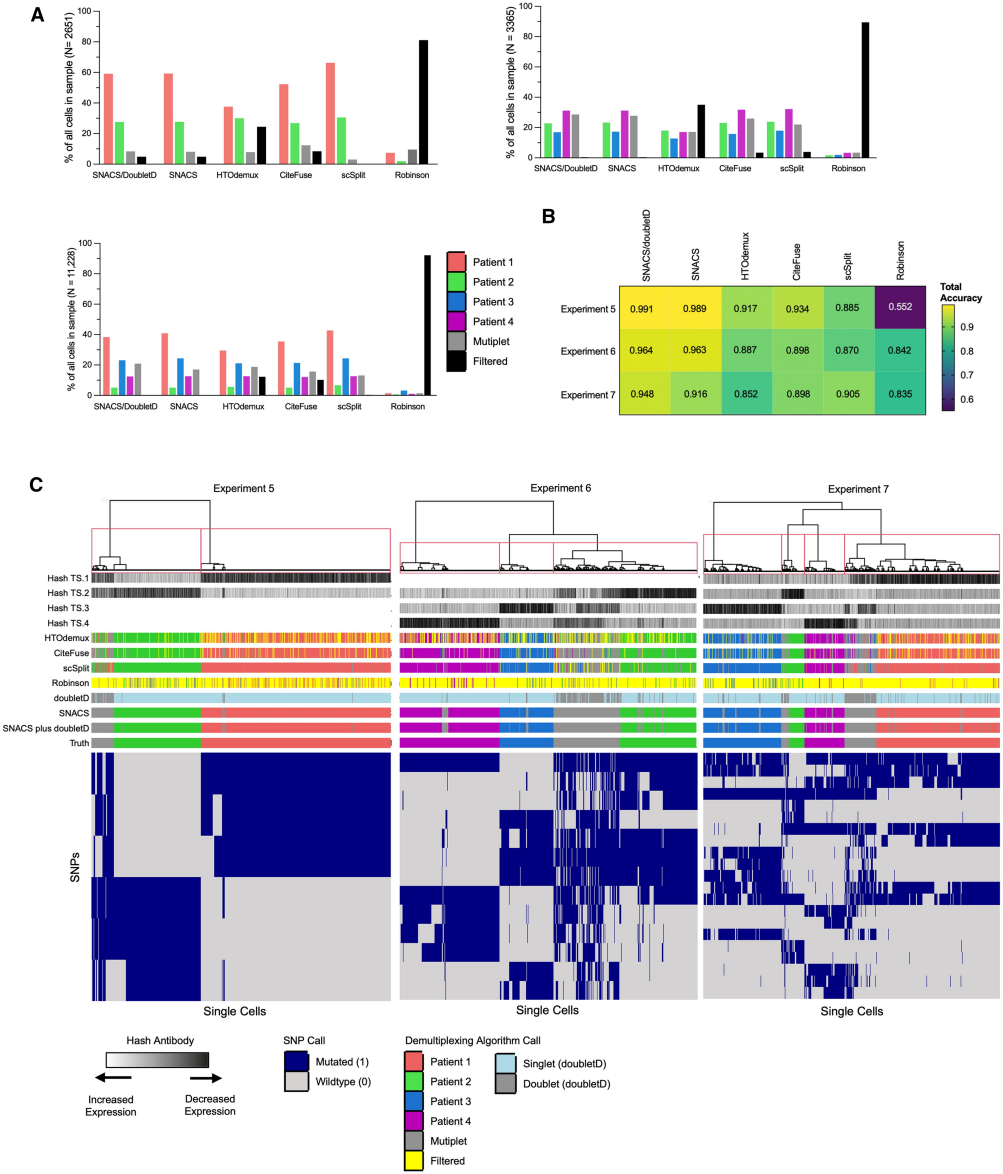
Comparison of SNACS to alternate demultiplexing algorithms. (A) Bar plots comparing relative proportions of singlets, multiplets, and filtered cells for SNACS plus alternative demultiplexing methods for multi-sample Experiments 5 (top left, patients A + B), 6 (top right, patients B + C + D), and 7 (bottom right, patients A + B + C + D). Relative to the comparison method s, SNACS filtered fewer cells. (B) Heatmap of total accuracy for SNACS plus alternative demultiplexing methods (columns) for multi-sample Experiments 5–7 (rows). (C) Heatmap of single cells (columns) versus SNPs used in final clustering by SNACS (rows) for multi-sample Experiments 5, 6, and 7. Rows at the top of the heatmap represent, in order from top to bottom, hash antibody signals; sample calls by HTOdemux, CiteFuse, scSplit, the Robinson method, doubletD, SNACS, SNACS plus doubletD, and truth calls.

We evaluated the performance of SNACS using empiric data from scDAb-seq of leukemia patients as outlined above. SNACS was able to accurately demultiplex multiplexed samples and identify multiplets (Fig. 2; Table 2; Tables S2–S4, available as supplementary data at *Bioinformatics* online).

Experient 5 contained multiplexed samples from two individual patients. Of the 2651 input cells, 2521 (95.1%) had sufficient genotyping information to be included in the SNACS algorithm. Of the 70 476 unique input SNPs, SNACS identified a total of 6 unique SNPs that best defined the initial antibody-based classifications and were used in subsequent hierarchical clustering (Table S5, available as supplementary data at *Bioinformatics* online). Of the 2651 single cells, SNACS assigned 1585 (59.8%) to Patient A, 735 (27.7%) to Patient B, and 200 (7.5%) as multiplets. When compared against the truth assessments, SNACS provided a total accuracy of 0.991, a sensitivity (called multiplets/true multiplets) of 0.980, and a specificity (called singlets/true singlets) of 0.992. With the addition of doubletD, sensitivity improved to 0.985, while specificity and total accuracy decreased to 0.990 and 0.981, respectively.

Experiment 6 contained multiplexed samples from three individual patients. Of the 3365 input cells, nearly all (99.6%) had sufficient genotyping information and were included in downstream analysis. SNACS assigned 799 (23.7%) to Patient A, 576 (17.1%) to Patient B, 1110 (33.0%) to Patient C, and 849 (25.2%) as multiplets. When compared against the truth assessments, SNACS provided a total accuracy of 0.964, a sensitivity of 0.976, and a specificity of 0.959. With the addition of doubletD, sensitivity again improved marginally to 0.992, while specificity decreased to 0.953.

Finally, Experiment 7 contained multiplexed samples from four individual patients. Of the 11 228 input cells, all (100%) had sufficient genotyping information and were included in downstream analysis. SNACS assigned 3753 (33.4%), 490 (4.4%), 2822 (25.1%), and 1452 (12.9%) to Patients A, B, C, and D, respectively; 1588 (14.1%) were assigned as multiplets. SNACS provided a total accuracy of 0.948, sensitivity of 0.919, and specificity of 0.953. Like Experiments 5 and 6, the addition of doubletD improved sensitivity (0.948) while decreasing specificity (0.910).

Crucially, across all three multi-sample experiments, SNACS very rarely assigned singlets to incorrect parent samples, occurring in 0% of cells in Experiment 5, 0.04% of cells in Experiment 6, and 0.02% of cells in Experiment 7.

### 3.2 Comparison of SNACS versus alternate barcode- and SNP-based approaches on empiric patient data

We also compared SNACS to 4 demultiplexing approaches and 1 doublet identification tool as outlined in section 2.1.3. Relative to these comparison methods, SNACS preserved a high number of single cells, with < 5% of input cells filtered in Experiment 5 and <1% in Experiments 6 and 7 compared to 0.15%–81.1%, 3.5%–89.5%, and 0.4%–92.2% for the same experiments with the comparison methods (Fig. 2A, Table 3). There was only one instance across methods and experiments (scSplit applied to Experiment 5) where an alternative method filtered fewer cells than SNACS.

**Table 3.** Accuracy metrics for SNACS and other demultiplexing approaches on empiric patient data.^a^

Algorithm	% cells filtered	Total accuracy	Sensitivity	Specificity	No. of true singlets	No. of true multiplets	Fraction of called singlets which are true alternative singlets
**Experiment 5 (*N* = 2651 cells)**							
SNACS	4.90%	**0.991**	0.980	**0.992**	2320	200 (7.5%)	0.000
SNACS or doubletD	4.90%	0.989	**0.985**	0.990	2320	200	0.000
HTOdemux	24.44%	0.917	0.581	0.979	1692	310	0.005
CiteFuse	8.49%	0.934	0.822	0.971	2099	326	0.001
scSplit	**0.15%**	0.885	0.187	0.984	2319	327	0.008
Robinson	81.10%	0.552	0.969	0.526	468	32	0.000
doubletD	0.0%	0.978	0.942	0.983	2320	330	N/A
**Experiment 6 (*N* = 3365 Cells)**							
SNACS	0.446%	**0.964**	0.976	**0.959**	2484	837 (25%)	0.0004
SNACS or doubletD	0.446%	0.963	**0.992**	0.953	2484	837	0.0004
HTOdemux	35.00%	0.887	0.758	0.905	1617	561	0.005
CiteFuse	3.45%	0.898	0.828	0.922	2408	812	0.004
scSplit	3.95%	0.870	0.719	0.916	2455	752	0.004
Robinson	89.48%	0.842	0.790	0.863	249	105	0.000
doubletD	0.0%	0.919	0.771	0.970	2485	849	N/A
**Experiment 7 (*N* = 11 228 Cells)**							
SNACS	0.0%	**0.948**	0.919	**0.953**	8517	1588 (14%)	0.0002
SNACS or doubletD	0.0%	0.916	**0.948**	0.910	8517	1588	0.0002
HTOdemux	12.31%	0.852	0.759	0.872	7392	1558	0.015
CiteFuse	10.22%	0.898	0.771	0.925	7557	1562	0.008
scSplit	0.39%	0.905	0.677	0.948	8494	1573	0.009
Robinson	92.20%	0.835	0.705	0.845	776	61	0.003
doubletD	0.0%	0.917	0.876	0.923	8517	1588	N/A

^a^ Total Accuracy is defined as proprtion correctly called cells/all called cells. Sensitivity is defined as proportion of True Multiplets that were called Multiplets. Specificity is defined as proportion of True Singlets that were called Singlets. Accuracy, Sensitivity, and Specificity are only calculated for cells that were both not filtered by the demultiplexing algorithm and non-ambiguous by truth call. Bolded values reflect the best performing algorithms for total accuracy, sensitivity, and specificity.

SNACS also offered superior sensitivity and specificity in all experiments with sensitivities of 0.98, 0.98, and 0.92 relative to 0.19–0.97, 0.72–0.83, and 0.68–0.88 for Experiments 5, 6, and 7 for the comparison methods (Table 2). Similarly, the specificity of SNACS was 0.99, 0.96, and 0.95 compared to 0.53–0.98, 0.86–0.92, and 0.85–0.93 for Experiments 5, 6, and 7 for the alternate methods (Table 2). Finally, SNACS misidentified the lowest proportion of singlets as belonging to a true alternative parent sample across all three multi-sample experiments. After SNACS, there was no one demultiplexing approach that provided the best accuracy, sensitivity, and specificity metrics across all experiments. Interestingly, there was also not a consistent pattern of superior performance between SNP-based versus barcode-based algorithms (Fig. 2B).

When compared to the Robinson method, SNACS identified a relatively similar number of SNPs as contributing to final clustering with 7, 13, and 20 SNPs identified by SNACS versus 11, 5, and 16 SNPs identified by the Robinson method for Experiments 5, 6 and 7, respectively (Table S5, available as supplementary data at *Bioinformatics* online). By contrast, scType included 1142, 663, and 1620 SNPs as contributing to the initial clustering.

These SNPs had incomplete overlap, with SNACS and the Robinson method sharing 1, 3, and 5 SNPs and SNACS and scType sharing 5, 8, and 14 SNPs for Experiments 5, 6, and 7, respectively. Taken together, these data suggest that filtering SNP data through population-level databases may miss a large proportion of highly discriminatory SNPs.

### 3.3 Performance of SNACS in an independent empiric validation cohort

SNACS was initially developed and tested using the data from Experiments 5–7, as outlined above. However, given the marked genetic and clonal diversity across human leukemia samples, we next chose to evaluate SNACS using an independent cohort of empiric scDAb-seq data from four additional patients with leukemia using the same experimental approach as outlined in Section 2.2. To additionally assess how SNACS performed in demultiplexing a larger number of patient samples, our four validation experiments included two, four, and eight patients multiplexed together, with the eight-patient experiment repeated as a biologic replicate (Table 3). Single-sample experiments and associated accuracy metrics are not available for this validation cohort.

**Figure 3.**
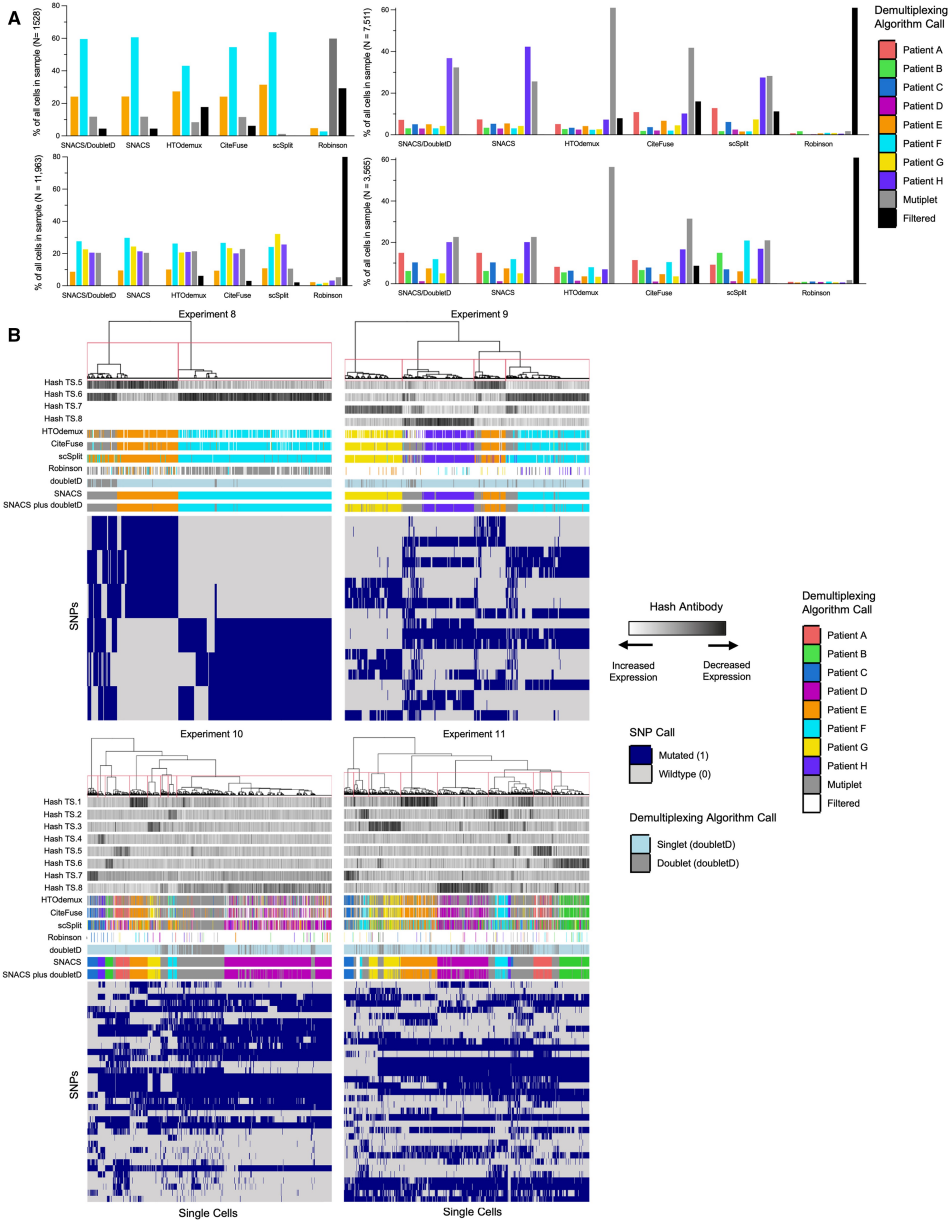
Comparison of SNACS to alternate demultiplexing algorithms in an independent validation cohort. (A) Bar plots comparing relative proportions of singlets, multiplets, and filtered cells for SNACS plus alternative demultiplexing methods for multi-sample Experiments 8 (top left, patients E + F), 9 (bottom left, patients E + F + G + H), 10 (top right, patients A + B + C + D + E + F + G + H) and 11 (bottom right, patients A + B + C + D + E + F + G + H, biological replicate of Experiment 10). (B) Heatmap of single cells (columns) versus SNPs used in final clustering by SNACS (rows) for multi-sample validation Experiments 8–11. Rows at the top of the heatmap represent, in order from top to bottom, hash antibody signals; sample calls by HTOdemux, CiteFuse, scSplit, the Robinson method, doubletD, SNACS, and SNACS plus doubletD.

Like the original cohort, our visualization demonstrates that SNACS was able to demultiplex multiplexed samples and identify multiplets, including for up eight multiplexed patients in two independent biologic replicates (Figs S6 and S7, available as supplementary data at *Bioinformatics* online). While accuracy metrics were not available for this cohort, our visualization also suggested that SNACS and SNACS plus doubletD again provided equivalent to superior performance relative to the comparison methodologies (Fig. 3A and B).

Relative to these comparison methods, SNACS filtered fewer cells apart from Experiment 9, in which fewer cells were filtered out by scSplit (Fig. 3A, Table S6, available as supplementary data at *Bioinformatics* online). Even with the lowest filtering rate, however, we found that when 8 samples were multiplexed, SNACS frequently called < 500 cells for an individual demultiplexed sample. For example, in Experiment 10, despite having 7511 input cells, SNACS called < 500 cells in 6 of 8 demultiplexed single samples. In Experiment 11, which had fewer input cells at 3565, SNACS again called < 500 cells in 6 of 8 demultiplexed single samples, and in one of those samples, only 44 cells were called. This finding raises concern that while demultiplexing > 4 samples may be informatically feasible, it may not be an appealing scientific strategy as it can result in a low number of cells per final demultiplexed sample.

### 3.4 Modeling impact of number of multiplexed samples, proportion of multiplets, number of input cells using simulated data

Using the simulated datasets described above, we assessed the ability of SNACS, SNACS + doubletD, and all comparison methods to demultiplex data consisting to 2, 3, and 4 samples multiplex; 2000, 4000, 6000, 8000, and 10 000 input cells, and 0.0, 0.5, 0.10, 0.15, 0.20, and 0.25 proportion multiplets; each simulation was repeated 100 times.

**Figure 4.**
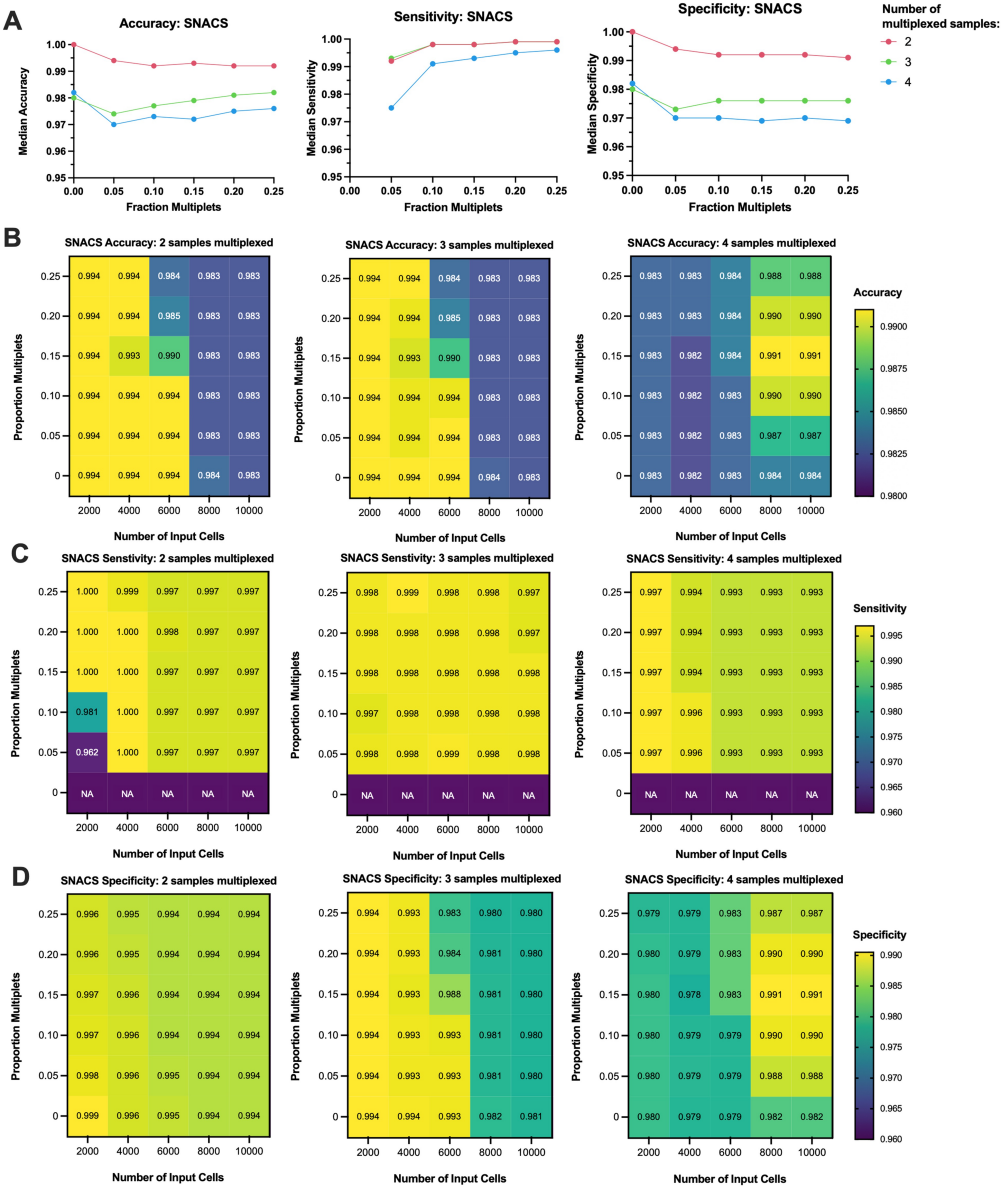
Performance of SNACS + doubletD on simulated datasets. Simulations were performed for 2000, 4000, 6000, 8000, and 10,000 input cells; 0.0, 0.05, 0.10, 0.15, 0.20, and 0.25 proportion multiplets; 2, 3, and 4 multiplexed samples. 100 replicates were performed for each condition. (A) Median accuracy (left), sensitivity (center), and specificity (right) vs proportion of multiplets for simulations using 2, 3, and 4 multiplexed samples across the full range of input cells. (B) Heatmap of median accuracy as impacted by proportion of multiplets (rows) and number of input cells (columns) for 2 (left), 3 (center), and 4 (right) multiplexed samples. (C) Heatmap of median sensitivity as impacted by proportion of multiplets (rows) and number of input cells (columns) for 2 (left), 3 (center), and 4 (right) multiplexed samples (D) Heatmap of median specificity as impacted by proportion of multiplets (rows) and number of input cells (columns) for 2 (left), 3 (center), and 4 (right) multiplexed samples.

SNACS performed robustly on all simulated datasets (Table S7, available as supplementary data at *Bioinformatics* online; Fig. 4). Accuracy remained high, at a median of 0.993 (5th and 95th percentiles: 0.974, 1.0), 0.978 (0.827, 0.998), and 0.973 (0.845, 0.998) for 2, 3, and 4 samples multiplexed, respectively, across the range of number of input cells and proportion of multiplets assessed. Similarly robust results were observed with sensitivity with a median of 0.999 (5th and 95th percentiles: 0.967, 1.0), 0.998 (0.987, 1.0), and 0.993 (0.955, 0.999), and for specificity, with a median of 1 (5th and 95th percentiles: 0.972, 1.0), 0.998 (0.816, 0.998), and 0.999 (0.833, 0.999), for 2, 3, and 4 samples multiplexed (Fig. 4A**)**. The number of samples multiplexed has the strongest impact on accuracy metrics, with median accuracy decreasing from 0.993 to 0.978 to 0.973 for 2, 3, and 4 multiplexed samples, respectively. The number of input cells also impacted accuracy, albeit to a minimal degree. Across all conditions, as the number of input cells increased from 2000 to 10 000, median total accuracy decreased from 0.988 to 0.982. By contrast, the proportion of multiplets had no clear impact: as the proportion of multiplets increased from 0.0 to 0.15 to 0.25, median accuracy remained relatively stable at 0.984, 0.982, and 0.987, respectively (Fig. 4B). Taken together, this suggests that an experimenter could safely increase the number of input cells, a strategy commonly used to identify rare cell populations, without sacrificing accuracy metrics.

Mirroring our empiric data, total accuracy for SNACS + doubletD was slightly below SNACS at a median of 0.955 (5th and 95th percentiles: 0.939, 0.988), 0.972 (0.82, 0.996), and 0.970 (0.844, 0.997) for 2, 3, and 4 multiplexed samples (Fig. S8, available as supplementary data at *Bioinformatics* online). Sensitivity, however, outperformed SNACS with a median of 1.0 (5th and 95th percentiles: 0.994, 1.0), 1.0 (0.996, 1.0), and 0.999 (0.995, 1.0), again suggesting that SNACS + doubletD may offer a superior approach to multiplet detection. Importantly, both SNACS and SNACS + doubletD preserved the large majority of cells; across all conditions and replicates, a median of 0% (5th and 95th percentiles: 0%, 8.14%) of cells were filtered due to being un-callable by the SNACS algorithm (Fig. S9A, available as supplementary data at *Bioinformatics* online).

Relative to SNACS and SNACS + doubletD, accuracy metrics for the comparison metrics were inferior in nearly all instances (Figs S9–S13, available as supplementary data at *Bioinformatics* online). Across all conditions tested, the median accuracy, sensitivity, and specificity were consistently superior for SNACS (Fig. S9B–D, available as supplementary data at *Bioinformatics* online), again confirming the robust performance of SNACS across a range of possible conditions. In addition to lower performance metrics, the comparison methods also filtered a larger number of single cells (Fig. S9A, available as supplementary data at *Bioinformatics* online). Across all conditions and replicates, HTOdemux filtered 20%–34%, CiteFuse 7%–12%, scSplit 1%–9%, and the Robinson Method 78%–96%. The only method which rarely filtered fewer cells was scSplit for 0.0, 0.05, and 0.10 proportion of multiplets and 2 samples demultiplexed: in these instances, scSplit filtered 0.05%, 1.02%, and 1.75% of cells versus 0.07%, 1.58%, and 3.10% for SNACS. For all other conditions, SNACS preserved a greater number of single cells.

### 3.5 Modeling maximum number of multiplexed samples using simulated data

From our empiric validation experiments, described above, we noted that multiplexing more than four single samples frequently led to <500 cells in a single demultiplexed sample, a number likely too low for meaningful downstream biologic analyses. Using simulated data, we modeled the probability of obtaining 100, 200, 300, 400, and 500 as the minimum number of cells in a demultiplexed sample as a function of the number of samples multiplexed.

**Figure 5.**
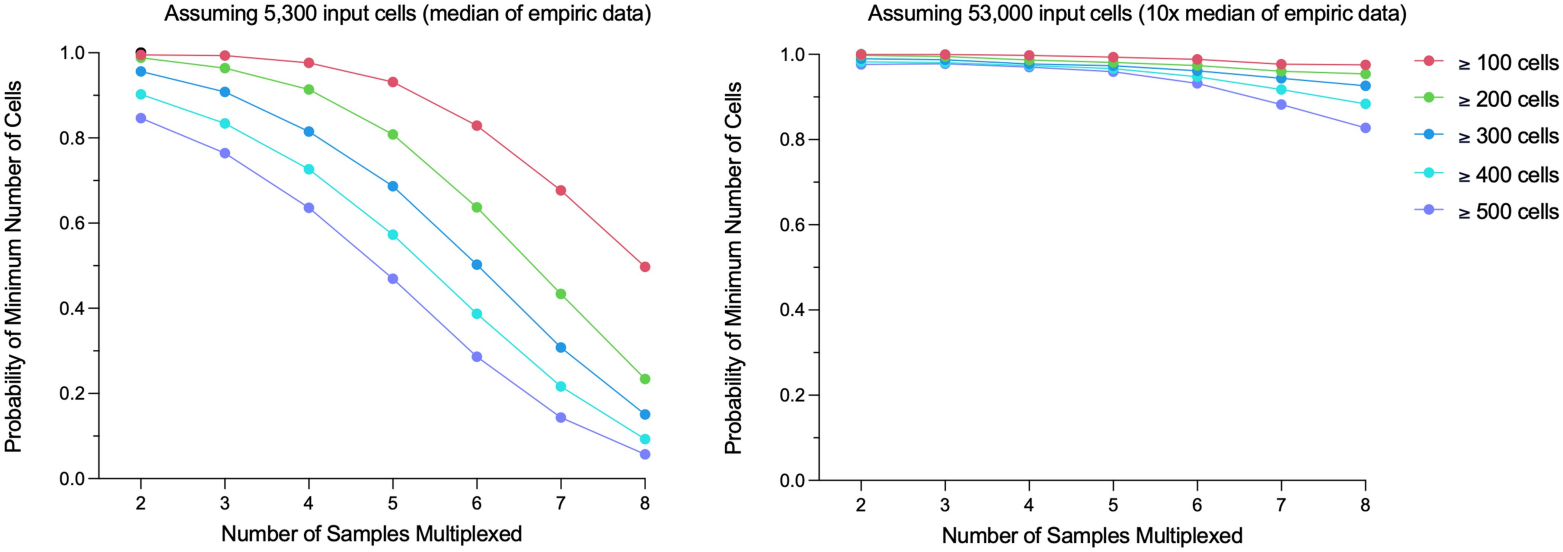
Modeling number of multiplexed samples using simulated data. Probability of obtaining at least 100, 200, 300, 400, and 500 as the minimum number of cells as a function of number of samples multiplexed. *Left*: Simulation performed using parameters derived from our 11 empiric experiments, including a mean of 5300 input cells with a standard deviation estimated at 2500 cells. *Right*: Simulation performed assuming the number of input cells could be increased 10-fold, now with a mean of 53 000 input cells with a standard deviation estimated at 25 000 cells.

Using our simulation model with an input of 5300 cells, the mean obtained in our 11 empiric experiments, we determined that multiplexing two samples together results in a probability of 0.847 of observing ≥ 500 cells per demultiplexed sample and a probability of 0.995 of observing ≥ 100 cells (Fig. 5). When the number of multiplexed samples was increased to four, the probability of observing ≥ 500 and ≥ 100 cells decreased to 0.636 and 0.976, respectively; when the number of multiplexed samples was increased to eight, the probability of observing ≥ 500 and ≥ 100 cells further decreased to 0.057 and 0.497.

Using our simulation model, this limitation can be overcome with a higher number of input cells. When we adjusted our simulation to parameters to assume a 10-fold increase in both cell input and standard deviation (i.e. to 53 000 and 25 000), the probability of observing ≥ 500 and ≥ 100 cells improved considerably, to 0.977 and 0.999 with two samples multiplexed, 0.970 and 0.998 with four samples multiplexed, and 0.827 and 0.976 with eight samples multiplexed (Fig. 5).

### 3.6 Tolerance for missing data

Due to variable cell fragility and viability, missing data is a common problem in assays involving primary samples from cancer patients. In our initial seven SNACS experiments, the percent of missing data ranged from 5.5% to 17.3%, and this missing data was imputed prior to clustering SNP data. To better understand the extent to which SNACS can tolerate missing data, for Experiments 5, 6, and 7 we randomly removed SNP data in increments of 5% until 50% of all data was missing, and then demultiplexed with SNACS as described above.

As expected, as the percent of missing data increased, the percent of total cells filtered increased, and the total accuracy decreased (Table S8, available as supplementary data at *Bioinformatics* online; Fig. S14, available as supplementary data at *Bioinformatics* online). For all three experiments, the percent of total cells filtered remained at <10% for up to 30%–40% missing data, then increased sharply (Fig. S14A, available as supplementary data at *Bioinformatics* online). Similarly, total accuracy remained > 0.90 for up to 40% missing data, then subsequently decreased (Fig. S14B, available as supplementary data at *Bioinformatics* online). Based on this model, we determined that SNACS performs optimally for missing data up to a threshold of ∼40%; for missing data greater than this, the filterData function in SNACS will issue a warning message.

## 4 Discussion

In recent years, the field of single-cell genomics has shifted from a handful of expert research laboratories to multiple research groups across diverse cancer histologies (Tanay and Regev 2017). A robust demultiplexing approach provides one avenue for scaling and democratizing this emerging technology. While demultiplexing approaches have been developed for scRNA-seq, the robust translation of these methods to scDNA-seq remains an unmet need. Here, we offer SNACS, a SNP-and-barcode-based algorithm for demultiplexing scDNA-seq data. SNACS is able to accurately assign singlets to parent samples without a priori knowledge of genetic features and, relative to existing approaches, provides greater sensitivity and specificity while simultaneously preserving a greater number of single cells. Finally, SNACS offers an accompanying data visualization, allowing the user to readily assess the quality of demultiplexing and decide whether additional optional multiplet refinement is appropriate for a particular dataset.

It is clear from our investigations that there is demultiplexing information in data from both natural genetic variation and in patient-level hash antibodies. We chose to combine these two data types in a particular way, but other combinations might be effective as well. Specifically, we utilized the hash data to choose an initial discriminatory set of SNPs, we clustered those SNPs, and we used the hash data to further divide cells with similar genotypes. If more cells were available for each genotype, we would explore independently classifying each genotype by its hash distribution. Since we are limited, our classification borrows strength across similar genotypes.

Our experimental patient data was obtained using the Mission Bio Tapestri bench-top machine and workflow and processed in an open source alignment pipeline (Demaree *et al.* 2021). SNACS is also compatible with genotype and antibody data outputted from the commercially available Mission Bio Tapestri alignment pipeline, although the SNP matrix must be formatted as binary (i.e. zygosity is not considered) and hash antibodies must first be normalized, such as by using the centered log ratio transformation. Finally, while we did not test SNACS using alternate scDNA-seq approaches, SNACS could potentially be applied to output from data generated by other scDNA-seq platforms provided the platform chemistry is compatible with cell-surface antibody tags and the input data is formatted correctly.

Our experimental data was derived from a targeted DNA panel using acute myeloid leukemia samples. This targeted panel includes genomic markers of greatest interest in leukemia, similar to what is commonly used in biological and clinical investigation and was not designed to include SNPs with maximal variation. SNACS is not limited to this histology and panel, however, and has been successfully used to demultiplex samples with mixed phenotypic acute leukemia sequenced with a different panel (Fig. S15, available as supplementary data at *Bioinformatics* online), thus allowing for biologically significant downstream analyses (Peretz *et al.* 2024). As the use of scDAbseq continues to expand, future work is needed to validate our approach across a range of histologies. Furthermore, while the panels used in our work provided adequate SNP coverage for accurate demultiplexing, it is possible SNACS and other barcode-based approaches could be further optimized through rational design of DNA panels to include genomic regions of high population-level variation. A future panel could be designed to include SNPs previously identified in population studies as maximally variable (Kayser and de Knijff 2011, Yousefi *et al.* 2018), or those commonly used in forensic analyses (Budowle and van Daal 2008, Tillmar *et al.* 2021). Y-chromosome encoded SNPs or short tandem repeat polymorphisms could also be included, and male and female patients could be intentionally multiplexed together (Jobling and Tyler-Smith 2017, Kayser 2017).

While our experimental data did not include additional cell-surface antibodies, these could be included in future experiments using SNACS, as binding of the hash antibodies used in this experiment does not interfere with binding of antibodies to other cancer-specific cell-surface antigens described in other scDNA-seq experiments.(Miles *et al.* 2020, Morita *et al.* 2020, Demaree *et al.* 2021). Furthermore, multiplexing multiple samples together could potentially mitigate batch effects, a particularly common problem with antibody staining, where subtle differences in antibody concentrations across samples can result in substantial apparent noise in raw protein counts, dominating over biological variations.(Stoeckius *et al.* 2017, Mule *et al.* 2022). SNACS, by allowing unique samples to be combined into one multi-sample experiment, minimizes batching and thus some batch effects.

While SNACS provides many strengths in an area of unmet need, there are limitations and tradeoffs to our approach that should be considered when it is used. Although accuracy was greater than against established methodologies, both in simulated data as well as empiric datasets, there were still a small proportion (<1.5%) of mis-classified singlets that were true multiplets. Should a biological experiment be conducted to identify small, rare cell populations, these mis-classified cells could result in false positive results. scDNA-seq experiments using SNACS will need to account for this, such as by discarding all cell populations smaller than a specific threshold. SNACS also requires that samples from different patients be multiplexed together. If an investigator wanted to analyze multiple timepoints from a single patient, samples from those timepoints would likely not be amenable to demultiplexing via SNACS.

SNACS is optimized to detect *across-sample* multiplets, in which multiplets are derived from different parent samples. In the single-cell literature, discussion also exists regarding *within-sample* multiplets, in which two or more cells from genotypically distinct subclones from the same parent sample are captured in a single droplet, resulting in a novel genotype derived from a single parent sample (Pellegrino *et al.* 2018, Kim *et al.* 2020, Weber *et al.* 2021). Unlike across-sample multiplets, within-sample multiplets may be present in single-sample experiments as well as multiplexed experiments. The true incidence of within-sample doublets in scDNAseq is unknown, and given their nature, they may not be detected by our truth-calling approach and algorithm. While SNACS does not directly detect within-sample multiplets, doubletD, which relies upon variant allele frequencies to detect multiplets, is optimized for both across- and within-sample doublets (Weber *et al.* 2021), and it is possible the “SNACS plus doubletD” output identifies within-sample multiplets as well. Indeed, the decreased sensitivity of “SNACS plus doubletD” may in fact reflect the identification of within-sample multiplets not identified by our truth calling approach.

Finally, questions remain regarding the number of patients that could successfully be multiplexed together. While our approach found a slight decrease in sensitivity and specificity with an increase from two to four multiplexed samples, we do not know whether this decrease would continue with increasing sample numbers, and if so, at what rate. Our validation experiments, which extended to eight multiplexed samples, did not include accuracy information; however, our visualization suggests that SNACS could be successfully used with a higher number of multiplexed samples. Rather than bioinformatic limitations, the ability to multiplex an increasing number of samples is more likely limited by technical considerations and the cell output of available scDNAseq platforms. In both our empiric and simulated data, we found that multiplexing beyond four samples frequently resulted in fewer than 500 cells per sample. While the optimal number of cells per patient is largely dependent upon the scientific or clinical research question of interest and the importance of detecting rare populations, the potentially small number of cells from a sample represents a practical limitation to our approach. As benchtop technologies continue to improve, we anticipate the ability to multiplex an increasing number of samples will increase as well.

## 5 Conclusion

Multiplexing scDNA-seq allows for efficient scaling of an emerging technology, and SNACS enables rapid and accurate demultiplexing and multiplet identification. Future studies are planned to validate and refine SNACS and evaluate it on larger numbers of multiplexed samples.

## Supplementary Material

btaf265_Supplementary_Data
