# Supplementary material for: SNACS: a tool for demultiplexing single-cell DNA sequencing data
Source: Bioinformatics. 2025 Jun 5;41(6):btaf265. doi: 10.1093/bioinformatics/btaf265 (PMC12208073; doi:10.1093/bioinformatics/btaf265)
Supplement: btaf265_Supplementary_Data [file btaf265_supplementary_data.zip › SNACSSupplementaryFigures_011625.pdf]

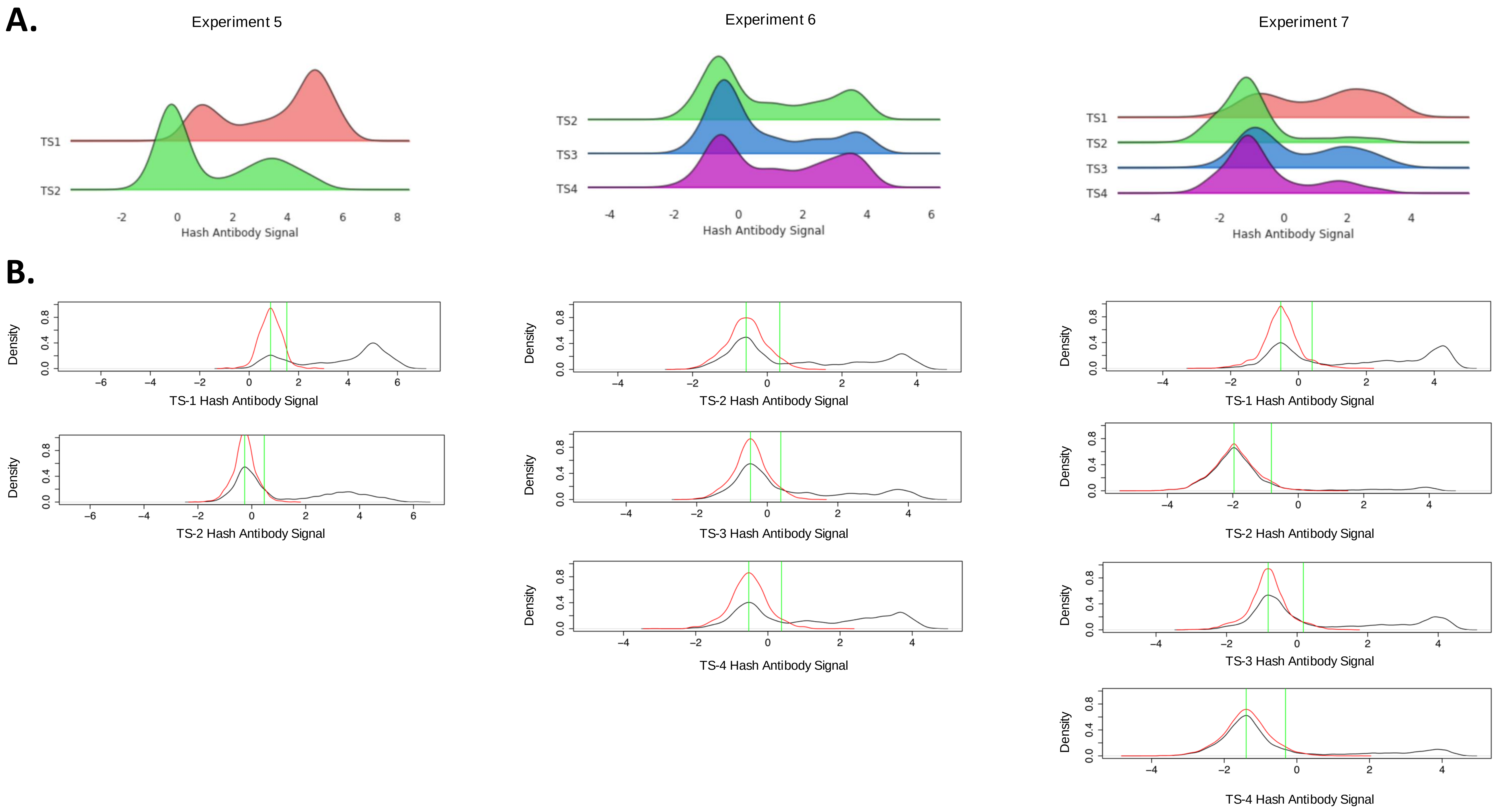

Supplemental Figure 1. Hash Antibody Distribution for Multi-Sample Experiments

A. Ridge plot of hash antibody expression for Experiment 5 (Patients A and B multiplexed), Experiment 6 (Patients B, C, and D multiplexed) and Experiment 7 (Patients A, B, C, and D multiplexed.

B. In the foundational SNACS algorithm, SNP-defined clusters are assigned to a specific hash by comparing the actual antibody expression of the cluster (black line) to the expected hash antibody distribution. To generate the expected bimodal hash antibody distribution**,** we fit a Gaussian distribution to the left-most actual distribution (red line) and reflect the data to the left of the mode about the mode.

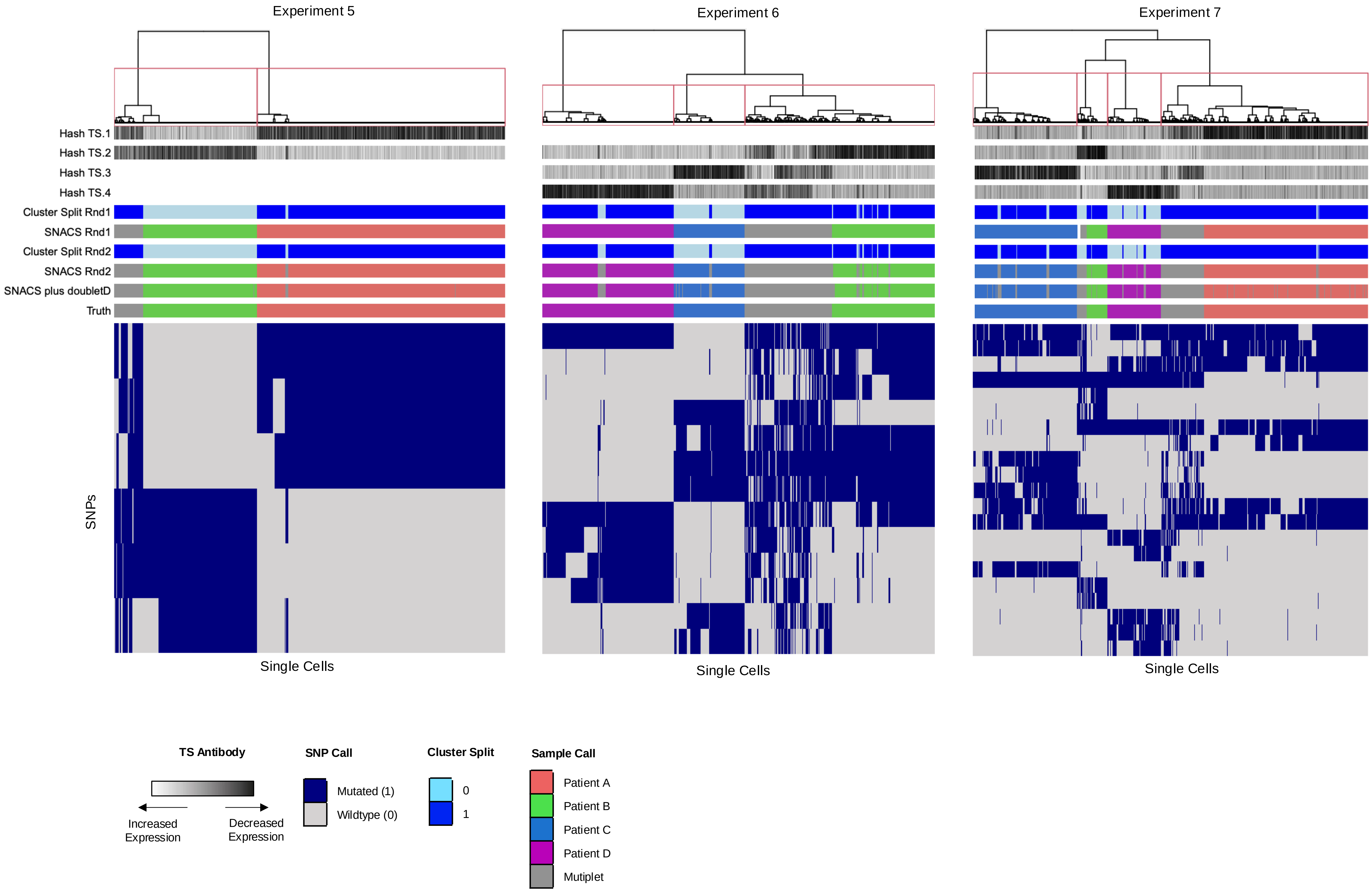

Supplementary Figure 2. SNACS offers simple visualization of demultiplexing algorithm as shown by heatmaps of multi-sample Experiment 5 (Patient A and B), Experiment 6 (Patient B, C, and D) and Experiment 7 (Patient A, B, C, and D.)
The heatmaps represents single cells (columns) vs SNPs (rows), color-coded by SNP mutational status. Rows above the heatmap represent, from top to bottom: hash antibody signal, cluster split and sample assignment from the foundational SNACs algorithm (SNACS Round 1), and cluster split and sample assignment from multiplet-refinement based on hash antibody signal (SNACS Round 2) and the inclusion of doubletD.

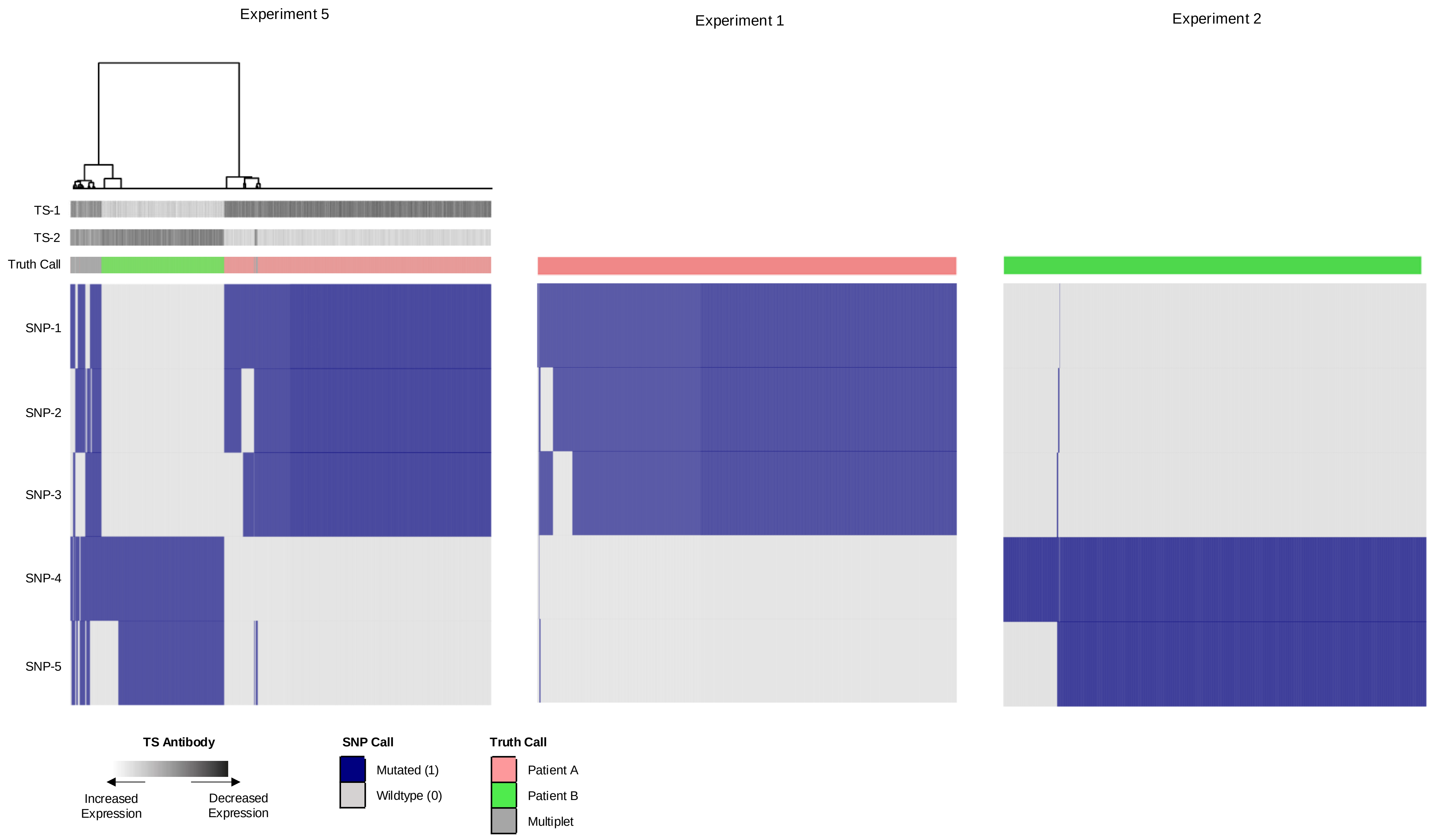

Supplemental Figure 3. Heatmaps of multi-sample Experiment 5 (Patients A and B Mutliplexed) and single-sample Experiments 1 and 2 (Patients A and B separately) provide visualization of accuracy assessment and calculation. The heatmap represents single cells (columns) vs SNPs (rows), color-coded by SNP mutational status. The 5 SNPs visualized are the SNPs which contributed to the final clustering in Experiment 5 which were also genotyped in Experiments 1 and 2. Rows above the heatmap represent, from top to bottom: hash antibody signal for TS-1 and TS2 and truth call as identified by single cell experiments.

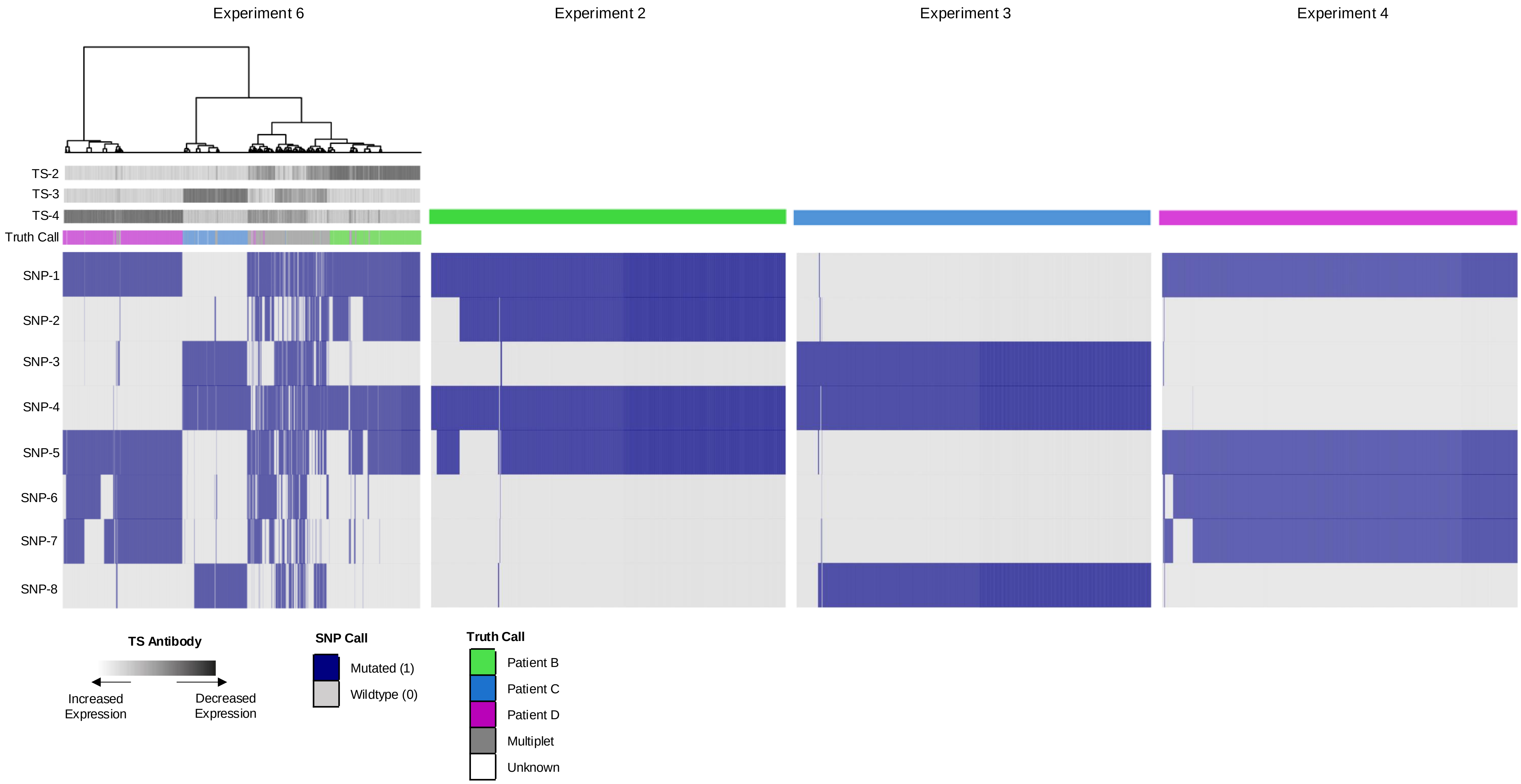

Supplemental Figure 4. Heatmaps of multi-sample Experiment 6 (Patients B, C, and D Mutliplexed) and single-sample Experiments 2, 3, and 4 (Patients B, C, and D, respectively) provide visualization of accuracy assessment. The heatmap represents single cells (columns) vs SNPs (rows), color-coded by SNP mutational status. The 7 SNPs visualized are the SNPs which contributed to the final clustering in Experiment 6 which were also genotyped in Experiments 2-4. Rows above the heatmap represent, from top to bottom: Hash antibody signal for TS-2, TS-3, and TS-4 and Truth Call as identified by single cell experiments.

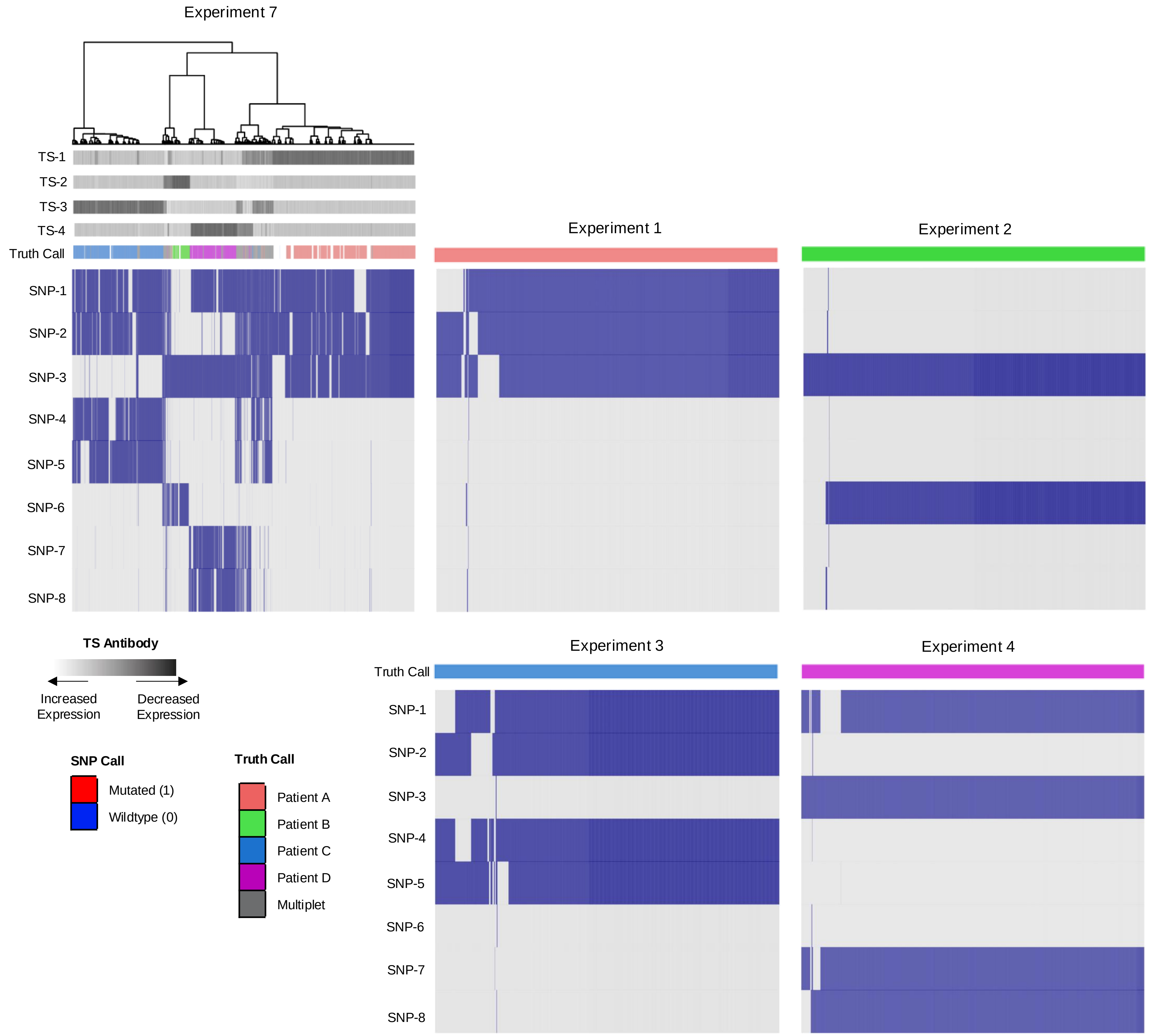

Supplemental Figure 5. Heatmaps of multi-sample Experiment 7 (Patients A, B, C, and D Mutliplexed) and single-sample Experiments 1, 2, 3, and 4 (Patients A, B, C, and D, respectively) provide visualization of accuracy assessment. The heatmap represents single cells (columns) vs SNPs (rows), color-coded by SNP mutational status. The 14 SNPs visualized are the SNPs which contributed to the final clustering in Experiment 7 which were also genotyped in Experiments 1-4. Rows above the heatmap represent, from top to bottom: Hash antibody signal for TS-1, TS-2, TS-3, and TS-4 and Truth Call as identified by single cell experiments.

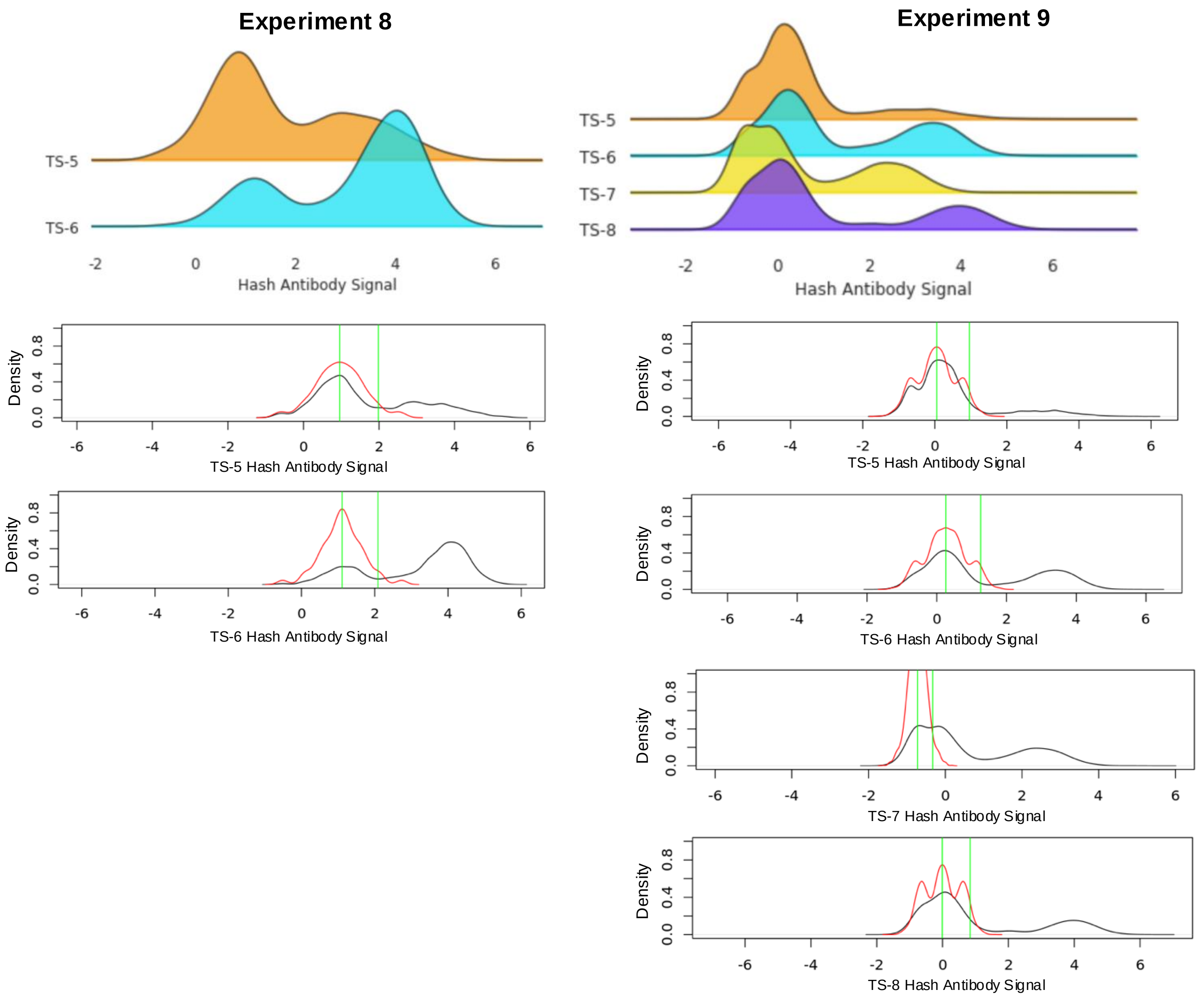

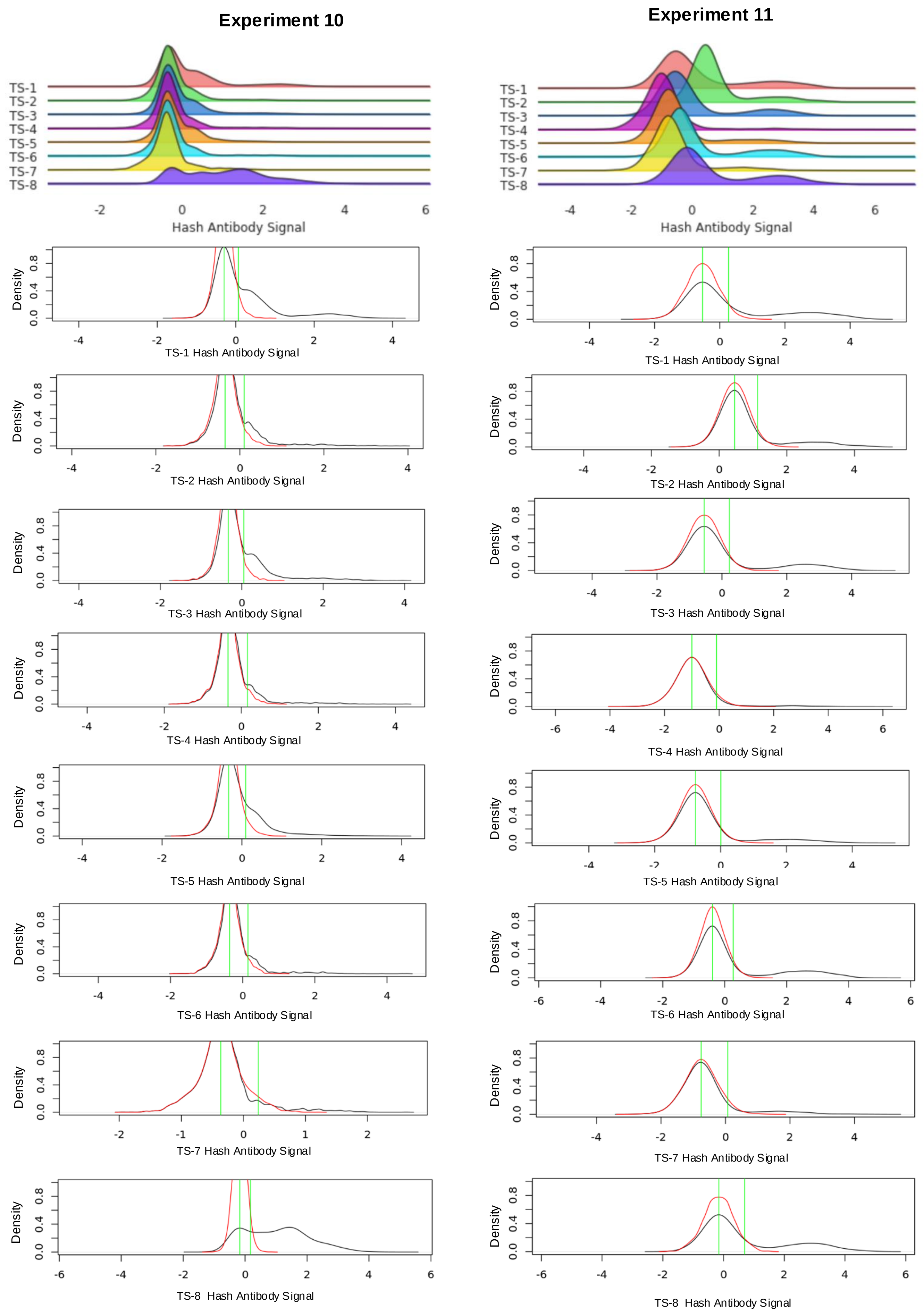

Supplemental Figure 6. Hash Antibody Distribution for Multi-Sample Experiments in Validation Experiments

(*Top)* Ridge plot of hash antibody expression for Experiment 8 (Patient E and F multiplexed), Experiment 9 (Patients E, F, G, and H multiplexed), Experiment 10 (Patients A, B, C, D, E, F, G, and H multiplexed), and Experiment 11 (Biological replicate of Experiment 10; (Patients A, B, C, D, E, F, G, and H multiplexed)

(*Bottom)* In the foundational SNACS algorithm, SNP-defined clusters are assigned to a specific hash by comparing the actual antibody expression of the cluster (black line) to the expected hash antibody distribution. To generate the expected bimodal hash antibody distribution**,** we fit a Gaussian distribution to the left-most actual distribution (red line) and reflect the data to the left of the mode about the mode.

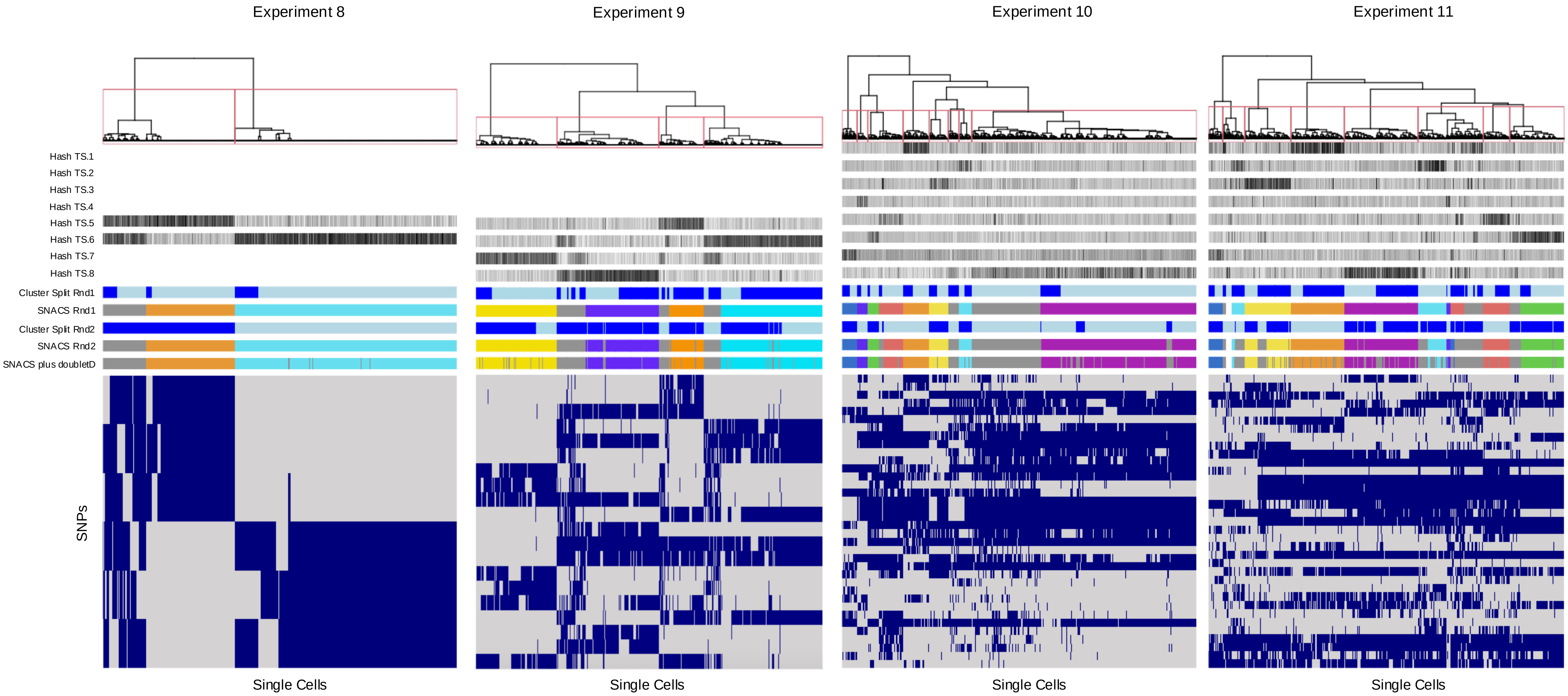

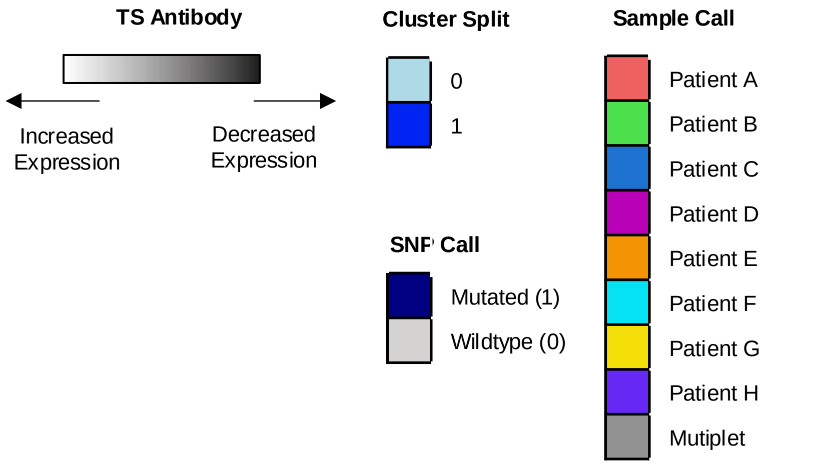

Supplementary Figure 7. SNACS offers simple visualization of demultiplexing algorithm as shown by heatmaps of multi-sample Experiments 8, 9, 10, and 11 from the validation cohort.

The heatmaps represents single cells (columns) vs SNPs (rows), color-coded by SNP mutational status. Rows above the heatmap represent, from top to bottom: hash antibody signal, cluster split and sample assignment from the foundational SNACs algorithm (SNACS Round 1), and cluster split and sample assignment from multiplet-refinement based on hash antibody signal (SNACS Round 2) and the inclusion of doubletD.

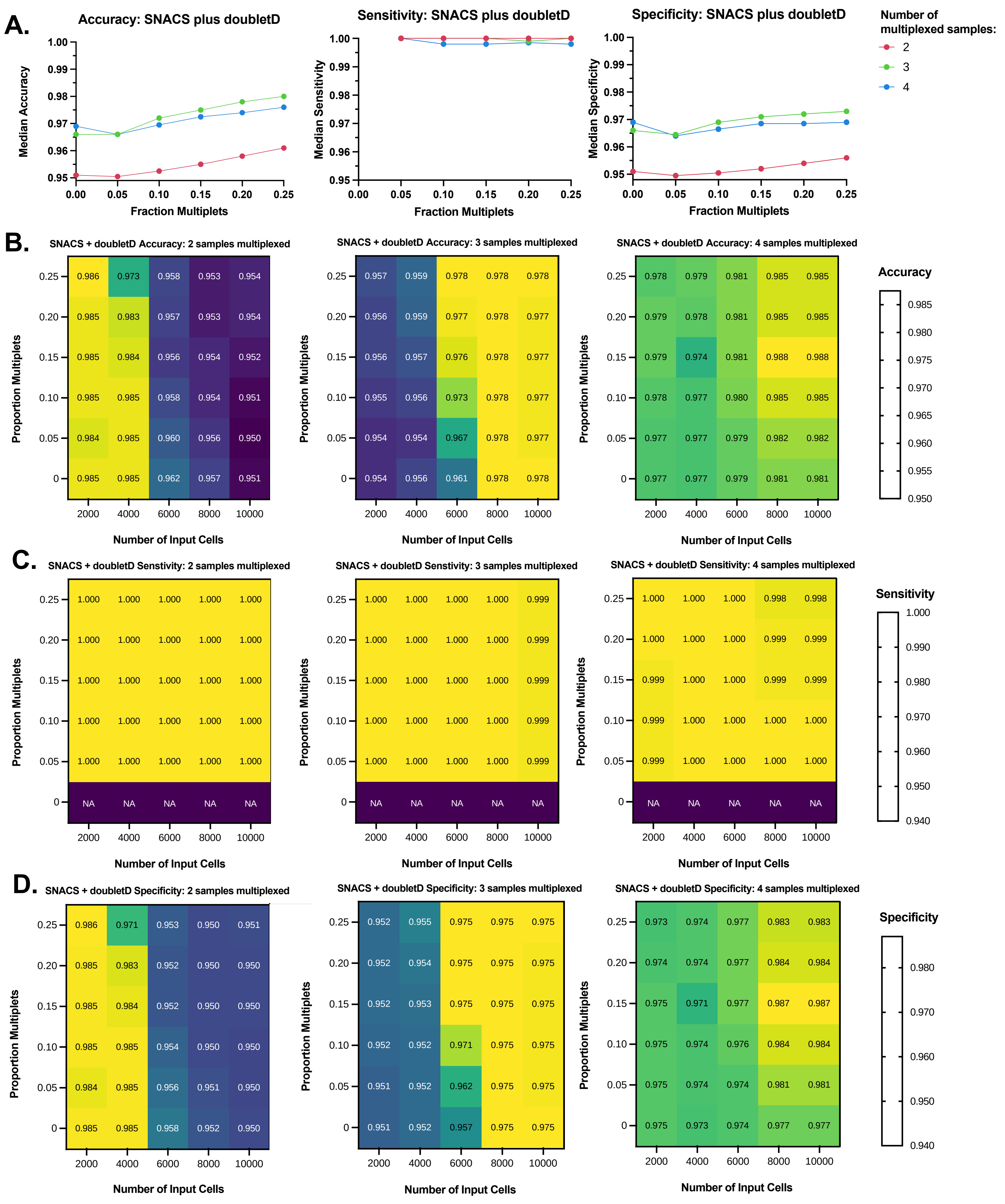

Supplementary Figure 8. Performance of SNACS + doubletD on simulated datasets. Simulations were performed for 2000, 4000, 6000, 8000, and 10,000 input cells; 0.0, 0.05, 0.10, 0.15, 0.20, and 0.25 proportion multiplets; 2, 3, and 4 multiplexed samples. 100 replicates were performed for each condition.
A. Median accuracy (*left),* sensitivity (*center)*, and specificity (*right*) vs proportion of multiplets for simulations using 2, 3, and 4 multiplexed samples across the full range of input cells.
B. Heatmap of median accuracy as impacted by proportion of multiplets (rows) and number of input cells (columns) for 2 (*left*), 3 (*center*), and 4 (*right*) multiplexed samples.
C. Heatmap of median sensitivity as impacted by proportion of multiplets (rows) and number of input cells (columns) for 2 (*left*), 3 (*center*), and 4 (*right*) multiplexed samples
D. Heatmap of median specificity as impacted by proportion of multiplets (rows) and number of input cells (columns) for 2 (*left*), 3 (*center*), and 4 (*right*) multiplexed samples

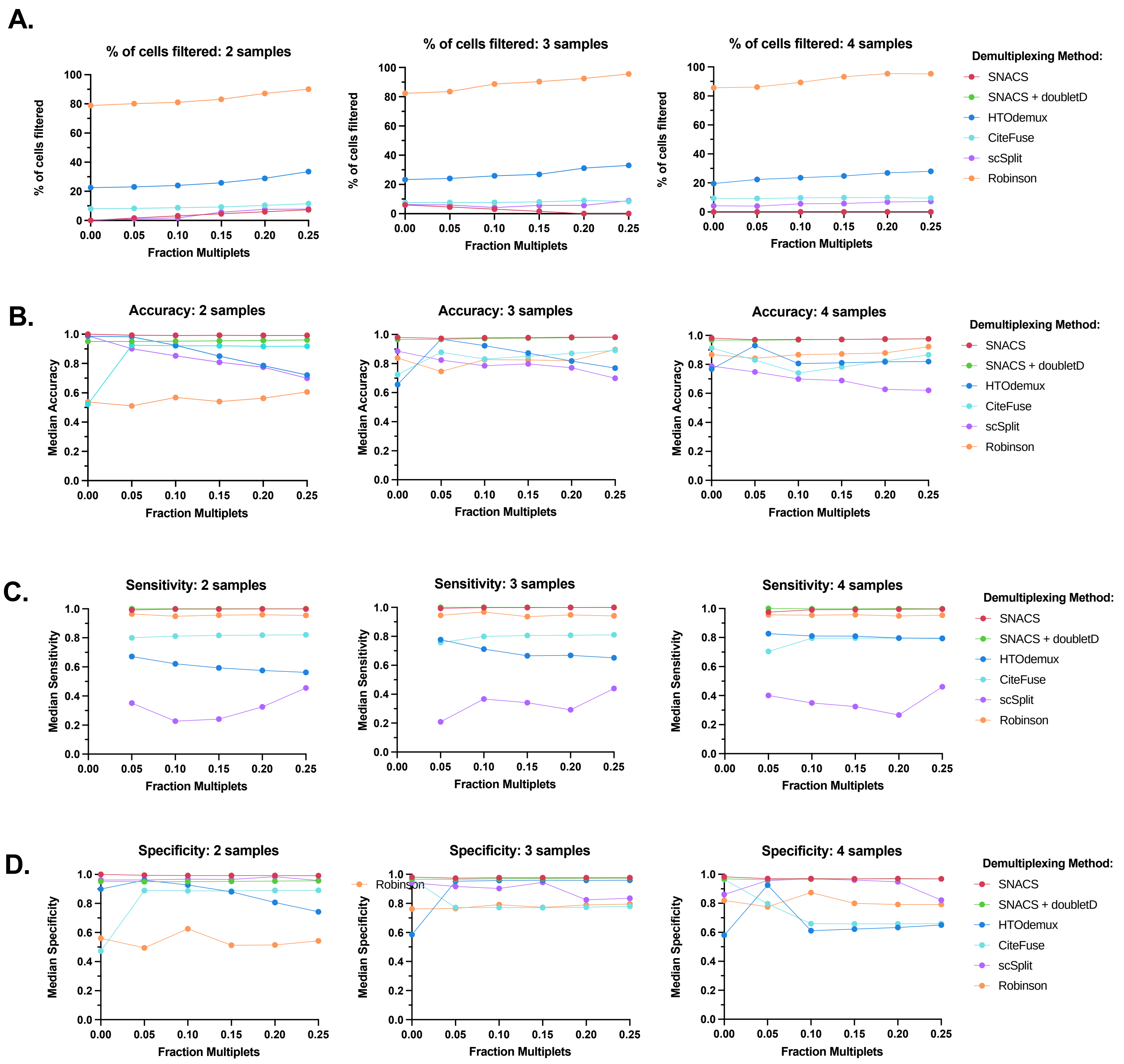

Supplementary Figure 9. Performance metrics for SNACS, SNACS + doubletD, and all comparison methods using simulated data. Simulations were performed for 2000, 4000, 6000, 8000, and 10,000 input cells; 0.0, 0.05, 0.10, 0.15, 0.20, and 0.25 proportion multiplets; 2, 3, and 4 multiplexed samples. 100 replicates were performed for each condition.

A. Median percent of cells filtered due to uncallable parent sample identity vs proportion multiplets for 2 (*left*), 3 (*center*), and 4 (*right*) samples multiplexed.

B. Median accuracy vs proportion multiplets for 2 (*left*), 3 (*center*), and 4 (*right*) samples multiplexed.

C. Median sensitivity vs proportion multiplets for 2 (*left*), 3 (*center*), and 4 (*right*) samples multiplexed.

D. Median specificity vs proportion multiplets for 2 (*left*), 3 (*center*), and 4 (*right*) samples multiplexed.

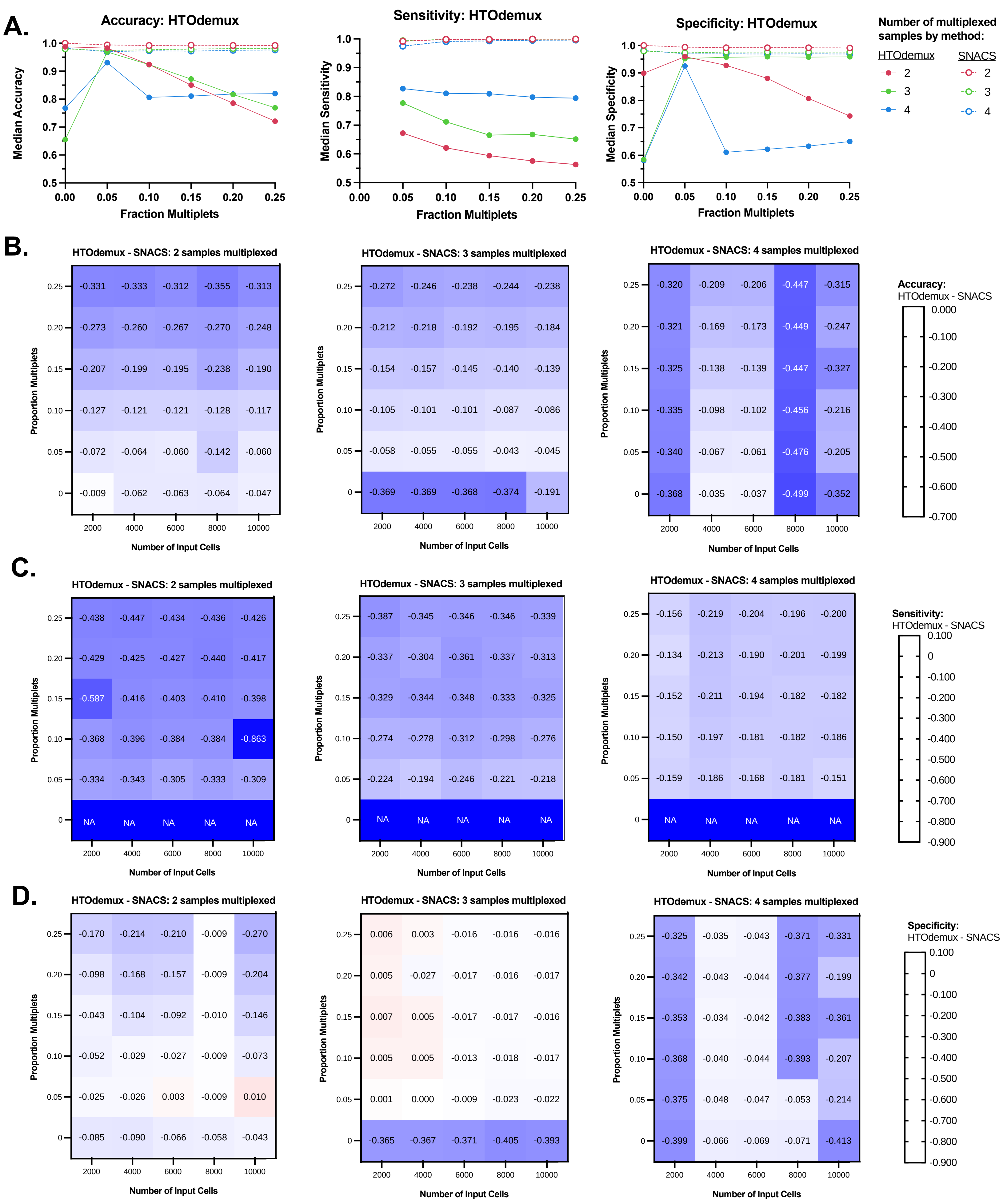

Supplementary Figure 10. Performance of HTOdemux on simulated datasets. Simulations were performed for 2000, 4000, 6000, 8000, and 10,000 input cells; 0.0, 0.05, 0.10, 0.15, 0.20, and 0.25 proportion multiplets; 2, 3, and 4 multiplexed samples. 100 replicates were performed for each condition.

A. Median accuracy (*left*), sensitivity (*center*), and specificity (*right*) vs proportion of multiplets for simulations using 2, 3, and 4 multiplexed samples across the full range of input cells. **HTOdemux** is represented by solid lines; SNACS is represented by dashed lines.

B. Subtraction heatmap of median accuracy of **HTOdemux** minus median accuracy of SNACS as impacted by proportion of multiplets (rows) and number of input cells (columns) for 2 (*left*), 3 (*center*), and 4 (*right*) multiplexed samples.

C. Subtraction heatmap of median sensitivity of **HTOdemux** minus median accuracy of SNACS as impacted by proportion of multiplets (rows) and number of input cells (columns) for 2 (*left*), 3 (*center*), and 4 (*right*) multiplexed samples.

D. Subtraction heatmap of median specificity of **HTOdemux** minus median accuracy of SNACS as impacted by proportion of multiplets (rows) and number of input cells (columns) for 2 (*left*), 3 (*center*), and 4 (*right*) multiplexed samples.

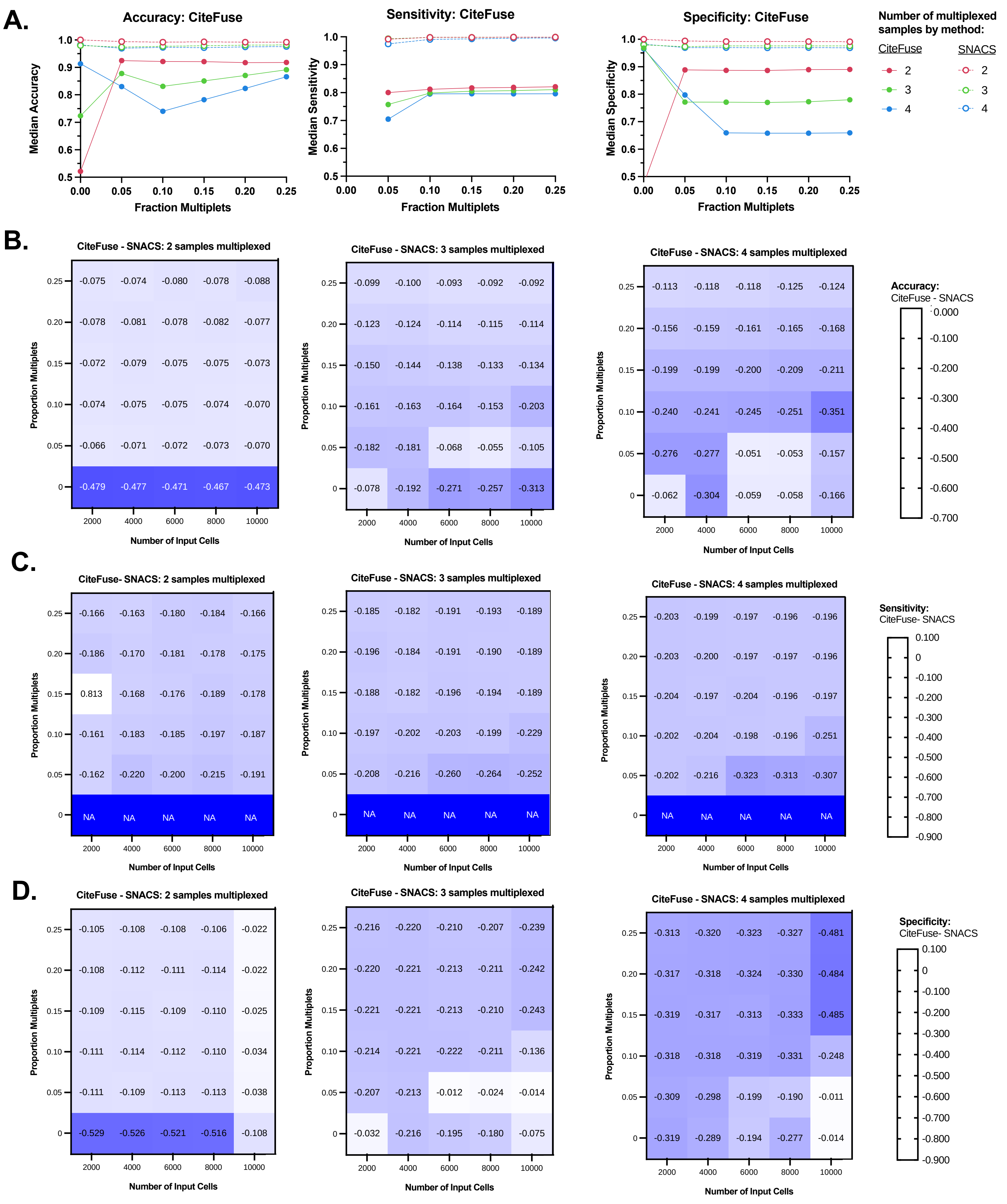

Supplementary Figure 11. Performance of CiteFuse on simulated datasets. Simulations were performed for 2000, 4000, 6000, 8000, and 10,000 input cells; 0.0, 0.05, 0.10, 0.15, 0.20, and 0.25 proportion multiplets; 2, 3, and 4 multiplexed samples. 100 replicates were performed for each condition.

A. Median accuracy (*left*), sensitivity (*center*), and specificity (*right*) vs proportion of multiplets for simulations using 2, 3, and 4 multiplexed samples across the full range of input cells. **CiteFuse** is represented by solid lines; SNACS is represented by dashed lines.

B. Subtraction heatmap of median accuracy of **CiteFuse** minus median accuracy of SNACS as impacted by proportion of multiplets (rows) and number of input cells (columns) for 2 (*left*), 3 (*center*), and 4 (*right*) multiplexed samples.

C. Subtraction heatmap of median sensitivity of **CiteFuse** minus median accuracy of SNACS as impacted by proportion of multiplets (rows) and number of input cells (columns) for 2 (*left*), 3 (*center*), and 4 (*right*) multiplexed samples.

D. Subtraction heatmap of median specificity of **CiteFuse** minus median accuracy of SNACS as impacted by proportion of multiplets (rows) and number of input cells (columns) for 2 (*left*), 3 (*center*), and 4 (*right*) multiplexed samples.

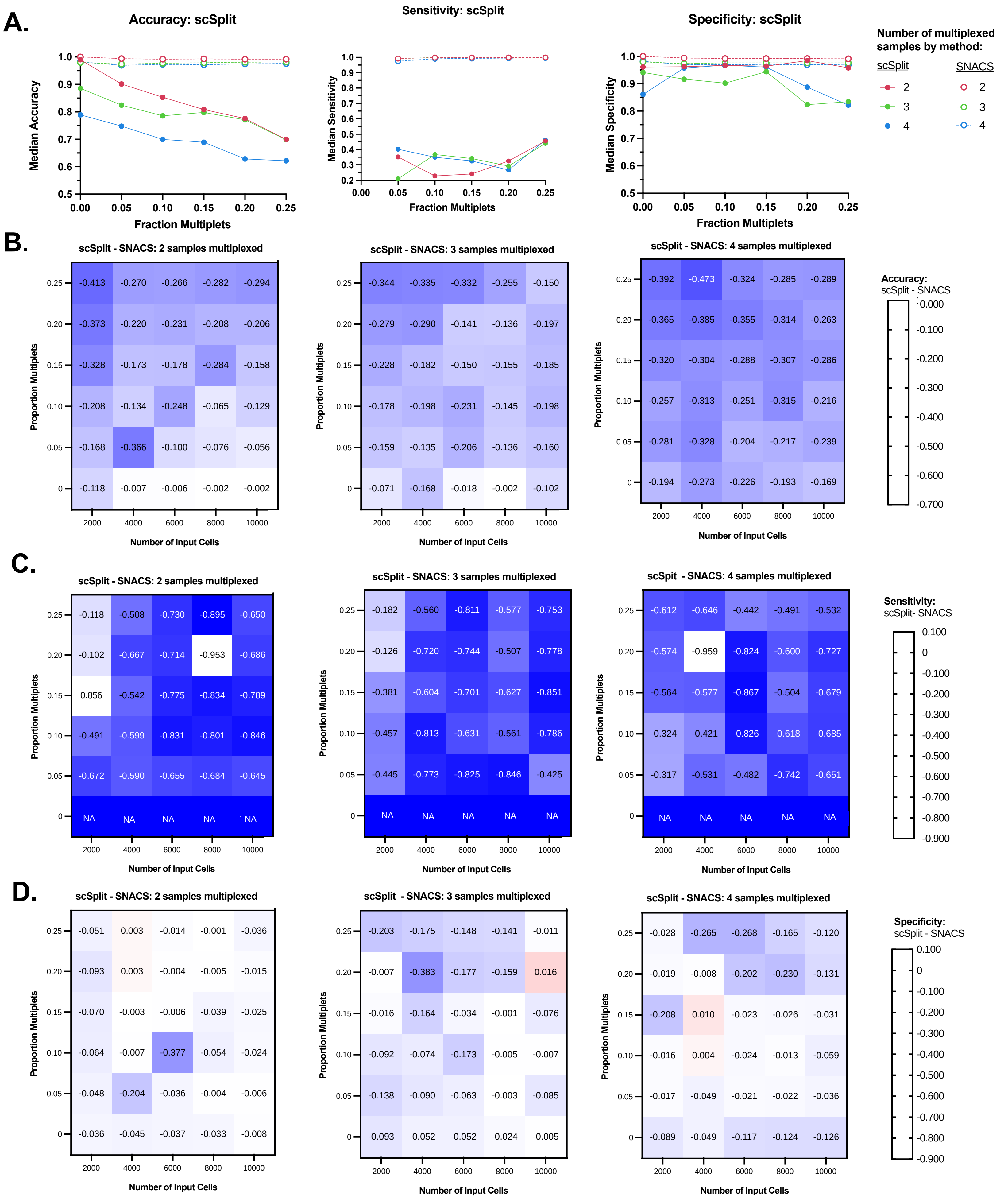

Supplementary Figure 12. Performance of scSplit on simulated datasets. Simulations were performed for 2000, 4000, 6000, 8000, and 10,000 input cells; 0.0, 0.05, 0.10, 0.15, 0.20, and 0.25 proportion multiplets; 2, 3, and 4 multiplexed samples. 100 replicates were performed for each condition.

A. Median accuracy (*left*), sensitivity (*center*), and specificity (*right*) vs proportion of multiplets for simulations using 2, 3, and 4 multiplexed samples across the full range of input cells. **scSplit** is represented by solid lines; SNACS is represented by dashed lines.

B. Subtraction heatmap of median accuracy of **scSplit** minus median accuracy of SNACS as impacted by proportion of multiplets (rows) and number of input cells (columns) for 2 (*left*), 3 (*center*), and 4 (*right*) multiplexed samples.

C. Subtraction heatmap of median sensitivity of **scSplit** minus median accuracy of SNACS as impacted by proportion of multiplets (rows) and number of input cells (columns) for 2 (*left*), 3 (*center*), and 4 (*right*) multiplexed samples.

D. Subtraction heatmap of median specificity of **scSplit** minus median accuracy of SNACS as impacted by proportion of multiplets (rows) and number of input cells (columns) for 2 (*left*), 3 (*center*), and 4 (*right*) multiplexed samples.

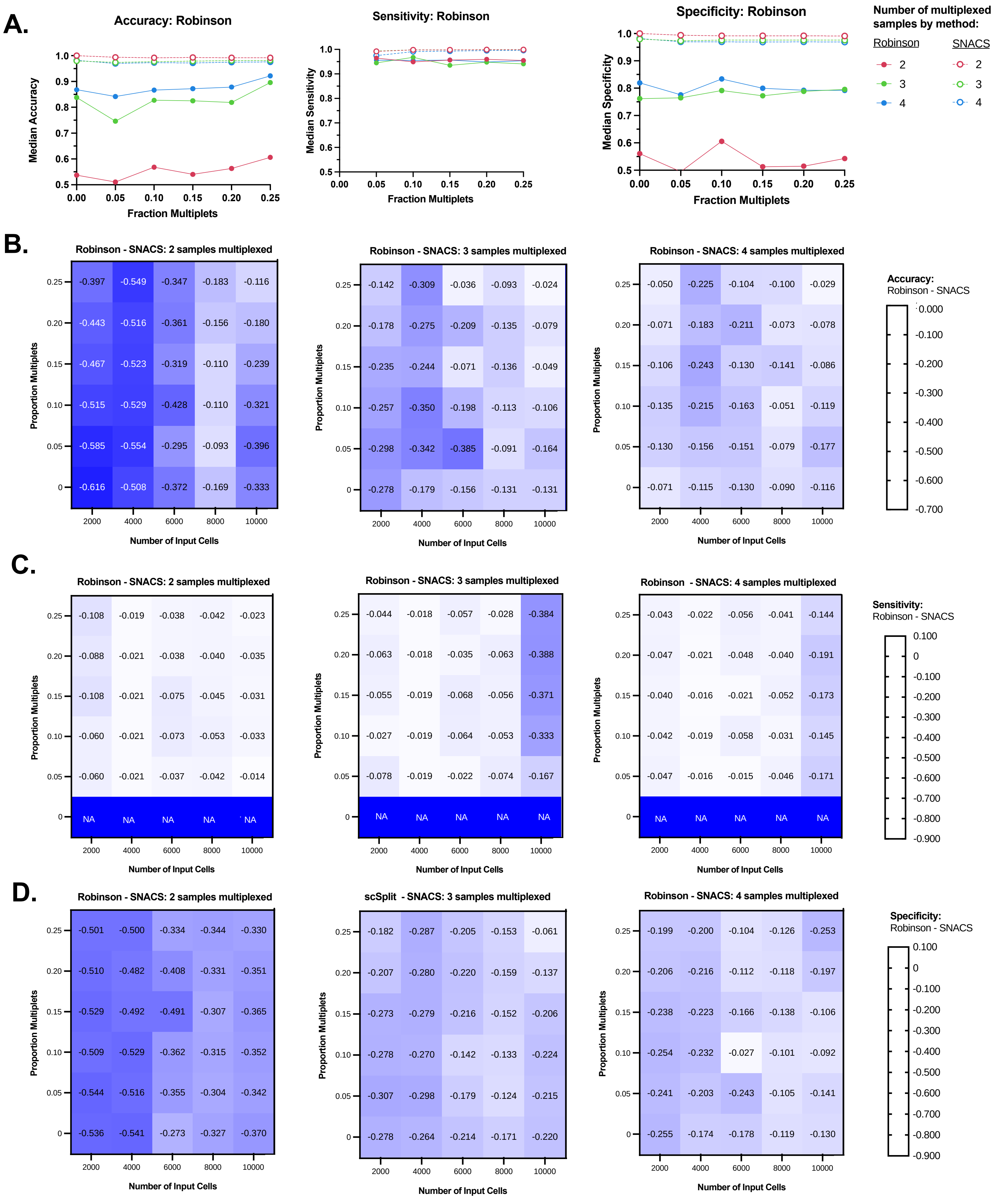

Supplementary Figure 13. Performance of Robinson method on simulated datasets. Simulations were performed for 2000, 4000, 6000, 8000, and 10,000 input cells; 0.0, 0.05, 0.10, 0.15, 0.20, and 0.25 proportion multiplets; 2, 3, and 4 multiplexed samples. 100 replicates were performed for each condition.

A. Median accuracy (*left*), sensitivity (*center*), and specificity (*right*) vs proportion of multiplets for simulations using 2, 3, and 4 multiplexed samples across the full range of input cells. **Robinson method** is represented by solid lines; SNACS is represented by dashed lines.

B. Subtraction heatmap of median accuracy of **Robinson method** minus median accuracy of SNACS as impacted by proportion of multiplets (rows) and number of input cells (columns) for 2 (*left*), 3 (*center*), and 4 (*right*) multiplexed samples.

C. Subtraction heatmap of median sensitivity of **Robinson method** minus median accuracy of SNACS as impacted by proportion of multiplets (rows) and number of input cells (columns) for 2 (*left*), 3 (*center*), and 4 (*right*) multiplexed samples.

D. Subtraction heatmap of median specificity of **Robinson method** minus median accuracy of SNACS as impacted by proportion of multiplets (rows) and number of input cells (columns) for 2 (*left*), 3 (*center*), and 4 (*right*) multiplexed samples.

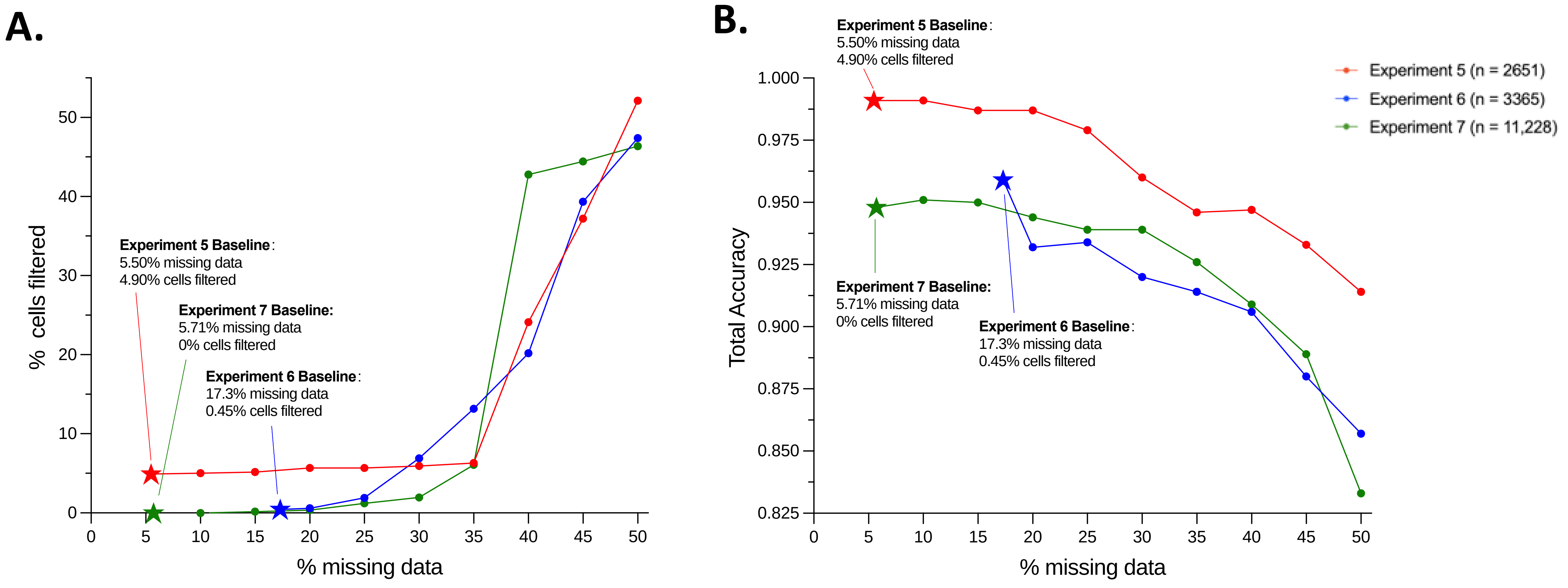

Supplementary Figure 14. Tolerance for Missing Data.

To model the effect of missing data on the performance of SNACS, for Experiments 5, 6, and 7 we randomly removed SNP data in increments of 5% until 50% of all data was missing, and then demultiplexed with SNACS.

A. Percent of total cells filtered vs % missing data for Experiment 5 (red; Patients A and B multiplexed), Experiment 6 (blue; Patients B, C and D multiplexed), and Experiment 7 (green; Patients A, B, C, and D multiplexed). Stars indicated the baseline missing data rate for each experiment.

B. Total Accuracy vs % missing data for Experiment 5 (red; Patients A and B multiplexed), Experiment 6 (blue; Patients B, C and D multiplexed), and Experiment 7 (green; Patients A, B, C, and D multiplexed). Stars indicated the baseline missing data rate for each experiment.

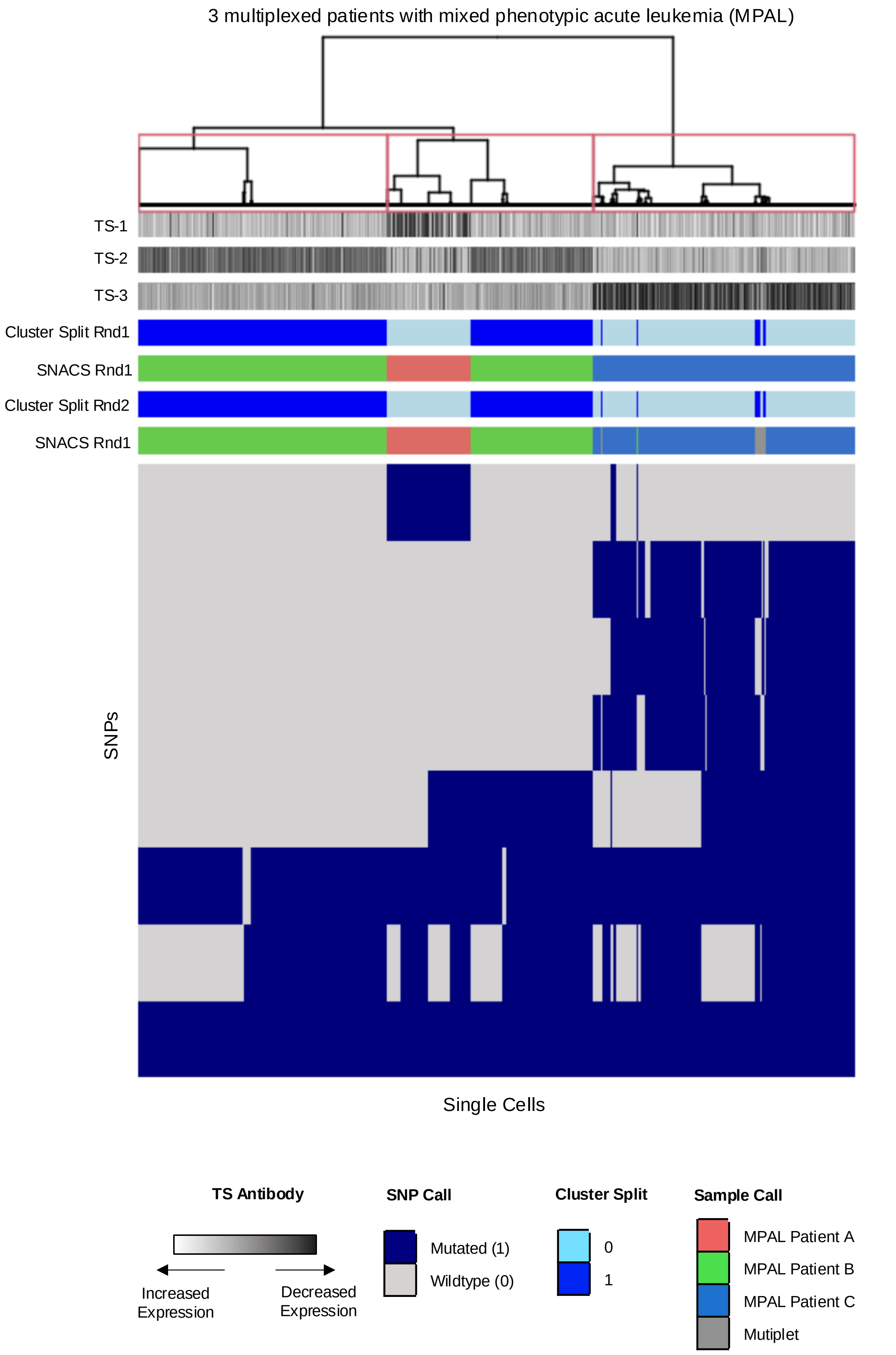

Supplementary Figure 15. SNACS Demultiplexing Visualization on 3 patients with mixed phenotypic acute leukemia (MPAL) who were sequenced on a distinct DNA panel. Downstream analysis of this demultiplexing was previously published[28]. This demonstrates the ability of SNACS to demultiplex various histologies and associated DNA sequencing panels.
